# Evaluating artisanal fishing of globally threatened sharks and rays in the Bay of Bengal, Bangladesh

**DOI:** 10.1371/journal.pone.0256146

**Published:** 2021-09-09

**Authors:** Alifa Bintha Haque, Rachel D. Cavanagh, Nathalie Seddon

**Affiliations:** 1 Department of Zoology, Nature-Based Solutions Initiative, University of Oxford, Oxford, United Kingdom; 2 British Antarctic Survey, Cambridge, United Kingdom; Institut de Recherche pour le Developpement, FRANCE

## Abstract

Sharks and rays are at risk of extinction globally. This reflects low resilience to increasing fishing pressure, exacerbated by habitat loss, climate change, increasing value in a trade and inadequate information leading to limited conservation actions. Artisanal fisheries in the Bay of Bengal of Bangladesh contribute to the high levels of global fishing pressure on elasmobranchs. However, it is one of the most data-poor regions of the world, and the diversity, occurrence and conservation needs of elasmobranchs in this region have not been adequately assessed. This study evaluated elasmobranch diversity, species composition, catch and trade within the artisanal fisheries to address this critical knowledge gap. Findings show that elasmobranch diversity in Bangladesh has previously been underestimated. In this study, over 160000 individual elasmobranchs were recorded through landing site monitoring, comprising 88 species (30 sharks and 58 rays) within 20 families and 35 genera. Of these, 54 are globally threatened according to the IUCN Red List of Threatened Species, with ten species listed as Critically Endangered and 22 species listed as Endangered. Almost 98% juvenile catch (69–99% for different species) for large species sand a decline in numbers of large individuals were documented, indicating unsustainable fisheries. Several previously common species were rarely landed, indicating potential population declines. The catch pattern showed seasonality and, in some cases, gear specificity. Overall, Bangladesh was found to be a significant contributor to shark and ray catches and trade in the Bay of Bengal region. Effective monitoring was not observed at the landing sites or processing centres, despite 29 species of elasmobranchs being protected by law, many of which were frequently landed. On this basis, a series of recommendations were provided for improving the conservation status of the elasmobranchs in this region. These include the need for improved taxonomic research, enhanced monitoring of elasmobranch stocks, and the highest protection level for threatened taxa. Alongside political will, enhancing national capacity to manage and rebuild elasmobranch stocks, coordinated regional management measures are essential.

## 1. Introduction

Elasmobranchs (sharks and rays) are the most threatened marine megafauna: around 36% face extinction, and 17% are Critically Endangered [1, 2], according to the International Union for Conservation of Nature (IUCN) Red List of Threatened Species (hereafter IUCN Red List) [3, 4]. A combination of factors has led to such high extinction risk e.g., relatively slow growth rate, low fecundity, and late age of maturity which result in low population recovery rates [5]. The high vulnerability to over-exploitation by by-catch and target fisheries together with habitat degradation have led to many of the world’s sharks and rays being threatened with extinction [6, 7–9]. As such, there is a global need for sustainable stock management and conservation [4, 10].

To implement effective management strategies, accurate identification with geographically appropriate taxonomic information, knowledge on diversity, seasonal occurrence, and gear specific catch patterns and trade are crucial [11–13]. Taxonomic information is crucial mainly because of inherent variation in biological characteristics among species influences their vulnerability. For instance, Rhinopristiformes rays (sawfish Pristidae, wedgefish Rhinidae, guitarfish Rhinobatidae, giant guitarfish Glaucostegidae) were identified to be the most threatened by many studies [14–16, 24] as opposed to many shark species capable of supporting sustainable fisheries [17]. Moreover, this specific information help contextualise the fishery problems that can differ geographically and where catch reports are patchy or conservative. Additionally, misidentified or aggregate catch reports are of limited use for designing effective conservation strategies [18]. As such, elasmobranch diversity needs to be well understood to appropriately assess the conservation needs against the exploitation of different species.

Due to difficulties in identifying many commonly fished elasmobranchs (e.g. carcharhinid, centrophorid, and triakid sharks, stingrays, skates, devil rays) [19–21], diversity in many parts of the world is undetermined. Given widespread taxonomic issues, sampling constraints and limited local expertise in many areas, there is a clear need for improved taxonomic studies, especially in the developing countries of the Indo-Pacific region [21–24]. Despite being a biodiversity hotspot, elasmobranch diversity of the Indo-Pacific is poorly known [21–24], especially in south-east Asia [6, 13, 25], including the Bay of Bengal region. The Bay of Bengal has a high elasmobranch species diversity, including endemic species, making it of high conservation importance. Moreover, a substantial proportion of taxa present here are genetically distinct from their closest relatives in other regions [26], bringing additional conservation challenges.

Limited knowledge of elasmobranch diversity and their particular threats, habitat use, catch and bycatch trend, is leading to depletion of several species with global conservation concern in the Bay of Bengal region (e.g. Ganges shark, giant guitarfish, and wedgefish, sawfish amongst many) [13, 24, 27, 28]. In addition, a historical baseline is lacking. Hence, the chances are high that several species are already depleted without being recorded or receiving any conservation or management actions [13, 29]. The scenario is exacerbated by the presence of the highest shark fishing and product exporter countries in this region (e.g. India) [1, 25, 30]. Indeed, India was one of the top shark fishing countries from 2007 to 2017, landing on an average of 73842 tonnes of sharks [25], contributing up to 9% of reported global landings [25, 31–33]. Although Bangladesh contributes significantly to the marine fisheries sector in the Bay of Bengal region, surveys regarding elasmobranchs in Bangladesh have been limited [34], with several questionable reports due to misidentified species or less knowledge on the range of these species [35, 36]. Only a few studies exist on the taxonomy and diversity of this group with sporadic catch pattern analysis and no or limited biological or ecological studies. Elasmobranchs were excluded from marine fisheries research for a long time due to difficulties in taxonomy, handling large specimens, resource constraints, and, most importantly, an underestimation of value in the formal marine fisheries sector, which has led to Bangladesh to be one of the most data-deficient countries globally.

To advance the understanding of the impacts of artisanal fishing on elasmobranchs in the Bay of Bengal, this study addressed critical knowledge gaps in evaluating the elasmobranch species composition (with correct and up-to-date taxonomy) of landings across key sites, including seasonal occurrence, distribution, and relative abundance, together with efficacy of gear type (i.e. to understand what gears are prone to more by-catch of elasmobranchs) and trade information. On this basis, the current status of the impacts of fisheries on elasmobranchs in the Bay of Bengal is discussed. The findings provide crucial information for conservation and management actions both in this region and globally, including Red List assessments and the UN Sustainable Development Goals (SDGs). Recommendations are made for conservation and management, as well as priorities for future work.

## 2. Materials and methods

### 2.1. Geographic context regarding threats and fisheries of elasmobranchs

The Bay of Bengal is a highly productive and heavily exploited ecosystem [37]. Due to the high productivity of this region, the historical fishing pressure has always been high with new and emerging fisheries [13]. Bay of Bengal is surrounded by eight developing countries (Bangladesh, India, Indonesia, Malaysia, Maldives, Myanmar, Sri Lanka and Thailand) with a high dependency on marine resources [38]. Hence, all these countries deploy an unprecedented number of fishing fleets to harness marine resources overexploiting the stocks for decades [39–41]. In addition to overfishing and harmful fishing techniques, such as industrial bottom and midwater trawling, Illegal, Unreported and Unregulated (IUU) fishing and high discard rates in different fisheries, other anthropogenic activities are problematic, including pollution (e.g. toxic run-off, heavy metal pollution and oil spills), harmful coastal agriculture and aquaculture practices, unregulated tourist activities (e.g. plastic pollution, light and noise pollution) and climate change [38, 42–61]. Documented impacts of these include habitat degradation, shifting spawning grounds, frequent coral bleaching [42–45], eutrophication, and a range of climate change impacts (e.g. sea-level rise, warming and ocean acidification) [47, 49]., all of which augment the problem, leading to shifting baselines and increasing the risk of stock collapse.

Bangladesh is situated at the northern tip of the Bay of Bengal. The dynamic coastline of Bangladesh comprises three major regions: the Ganges tidal plain in the west, which includes the Sundarbans Reserve Forest; the Meghna deltaic plain in the south-central region, and the Chittagong coastal plain in the east [62–64], along the coastline of 710 km [65]. The Sundarbans Reserve Forest lies within the Ganges-Brahmaputra delta in the Bay of Bengal, formed by the confluence of the Ganges, Padma, Brahmaputra, and Meghna rivers. It is the world’s largest contiguous halophytic mangrove forest, spanning 10000 km^2^, 62% in south-western Bangladesh, and the rest in India [66]. Its highly complex ecology includes freshwater, estuarine and marine habitats, thereby making it a unique habitat for many species [67], including elasmobranchs.

The fishing pressure in Bangladesh is substantially high [50]. The majority of the fishing in Bangladesh is conducted by artisanal fishers, employing gears including drift gill nets, set-bag nets, long lines, and trammel nets [68], targeting mostly hilsa, *Tenualosa ilisha* (Clupeidae) with some number of elasmobranchs either as by-catch or target (Haque in prep.). A total of 67669 boats with 188707 gear units are in operation in the coastal and marine waters with 247 industrial trawlers (in 2019) [50, 69]. There is substantial IUU fishing, in the form of under-reported commercial catch, discarded by-catch (e.g. sharks) [94] and subsistence catches [70], exploiting about a total of 442 species of fish and 915 species of other marine organisms which were reported from the coastal and marine waters [65].

Elasmobranchs are threatened in Bangladeshi waters due to substantial by-catch with unselected gears [Haque in prep., 35, 55], opportunistic catch and targeted ray fisheries. This is exacerbated by the existing international fin and meat trade [71] and poor landing monitoring mechanisms in place. Most significantly, they receive limited conservation actions due to data deficiency, lack of community awareness, facilitation in taking sustainable approaches, and finally, resource constraints. Bangladesh remains a conspicuous data gap regarding a comprehensive understanding of its elasmobranch diversity and catches despite being a highly fished region [41, 50, 70, 72–74].

The only established full record for elasmobranchs in Bangladesh is presented by the FAO report of the Bay of Bengal Large Marine Ecosystem Project until 2020 [34, 35] supplemented by a few research articles [74]. These are likely not entirely up to date. Morphological similarities and the presence of undescribed and cryptic species likely hampered identifications [75, 76] along with the incorporation of several species with geographical distributions reported outside of this region. The inadequate amount of directed research in elasmobranch diversity, distribution, and biology gives rise to scepticism about the comprehensiveness and precision of the available checklist. This has led to a limited assessment of species-specific vulnerability, which has contributed to uninformed protection of some species under national law regarding species protection (e.g. the Spadenose shark *Scoliodon laticaudus* is listed as Near Threatened on the IUCN Red List, yet is under national protection in schedule I (highest level of protection against catch and trade) [71], whereas the common shovelnose ray *Glaucostegus typus* is Critically Endangered on the IUCN Red List and listed in the Convention on International Trade in Endangered Species of Wild Fauna and Flora (CITES) App. II, yet not protected under national law).

### 2.2. Study sites

**Fig 1:**
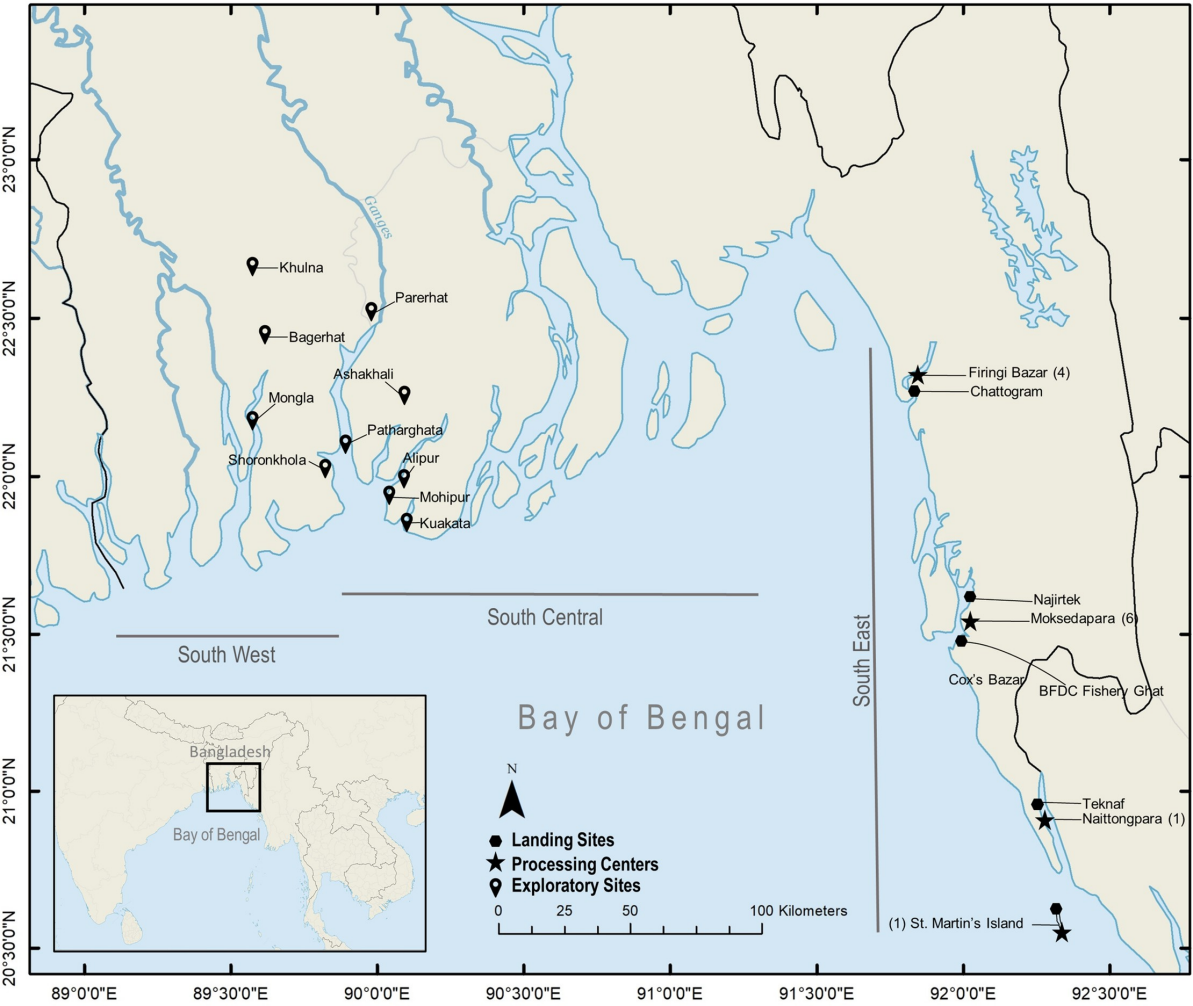
The inset map shows the location of Bangladesh in the Bay of Bengal. Map: The Bangladesh coastline showing the northern arm of the Bay of Bengal. Pinpoint icons show the exploratory field sites; hexagon icons indicate landings sites; and star icons indicate the processing centres, along the south-eastern coastline of Bangladesh.

Between January and November 2016, exploratory field visits in fourteen landing sites in three coastal regions of Bangladesh were conducted. They were: South-west (Khulna, Bagerhat, Mongla, Shoronkhola), South-central (Mohipur, Alipur, Parerhat, Patharghata, Ashakhali, Kuakata) and South-east (Chattogram, Cox’s Bazar, Teknaf, St. Martin’s Island) regions (Fig 1). These exploratory visits were conducted to identify the sites with the highest concentration of elasmobranch landings, processing centres and trade hubs. The south-east region was selected as this region is the hub for international elasmobranch trade [71], including 12 sizeable exclusive shark processing centres with substantially high production and trade capacity and contributing to landing from all other regions. This region was also identified as harbouring the biggest landing sites by volume of marine fish landing [70].

The current research project was primarily a fishery-dependent assessment enabling a comprehensive study in the south-east coastal region. The focus was on large landing sites that landed fish from vessels through-out the three zones. Small, informal landing sites were excluded from the study as the landing were negligible compared to the sites selected for ensuring better time efficiency covering the most landings within the study period.

### 2.3. Preparation of an annotated checklist

Before the field studies, an annotated checklist of all elasmobranchs reported from Bangladeshi waters was prepared from published documents found through a literature review [27, 34–36, 71–73, 77–89].

For this review, all available peer-reviewed articles from the Web of Science were collected using the search terms ‘Bay of Bengal* elasmobranchs’, ‘Bay of Bengal* elasmobranchs or sharks’, ‘Bangladesh* elasmobranchs or sharks or rays or batoids or sawfish’, Bangladesh* elasmobranchs’, Bangladesh* sharks and rays’, ‘Bangladesh* sharks’ and ‘Bangladesh* sharks or rays’; and reviewed. Government reports (Department of Fisheries, Fisheries Resource Survey System (FRSS) reports), Non-government Organisations (NGO), International Non-government Organisations (iNGO), the Bay of Bengal Large Marine Ecosystem (BOBLME) Project report, Indian Ocean Tuna Commission (IOTC) reports and other grey literature (newspaper articles) were searched from their websites, Google search engine and Google Scholar for completeness. Elasmobranch scientists in Bangladesh were personally contacted for any unpublished data or non-peer-reviewed works. Websites with global fisheries data [(e.g. Fishbase, Fishbase Bangladesh, the CITES trade database, the United Nations Commodity Trade Statistics Database (UN Comtrade)] were searched for additional information. Comments on previously misidentified reported species, possible occurrences, and species requiring further confirmation were made. Species have also been added to this list which considered as possible presence as the Bay of Bengal has been reported to be a range but was not yet reported in any national studies. The IUCN Red List assessment category, CITES, CMS and National protection statuses for each species are also listed. Validity status and occurrence from the region was confirmed and evaluated following recent publications and globally accepted range studies [87].

The checklist was modified after the field surveys conducted during 2015–2020 by the authors when a new record was made. Information shared by colleagues with evidences was also included when needed for completeness until December 2019.

### 2.4. Surveys

#### 2.4.1. Landing site and processing centre surveys

Between the 4th of January 2017 and the 30th of June 2017, surveys targeting elasmobranchs’ (classified as shark, Rhinopristiformes ray and other rays) morphometric data were conducted at landing sites for 15 days each month. Additionally, between 2018 and 2019, opportunistic landing data were collected specifically on the diversity of elasmobranchs. Large piles of landings comprising hundreds of small-sized rays were excluded from the study due to difficulties in accurately sampling these.

The number of elasmobranchs were counted in the landing sites. The range of the lengths of species landed was documented, and a sub-set of the counted individuals was measured for detailed biological parameters such as total length (TL) to the nearest cm and weighed (body weight, BW) in kg. TL for all specimens was measured when fins in caudal and/or tail parts were present, while BW could not be measured for several specimens because the specimens were too heavy and/or their fins had been cut. Photographs of all available whole-bodied elasmobranchs were taken for identification to the lowest possible taxonomic level using the keys of Compagno et al., 2005 and Last et al., 2016.

**Fig 2:**
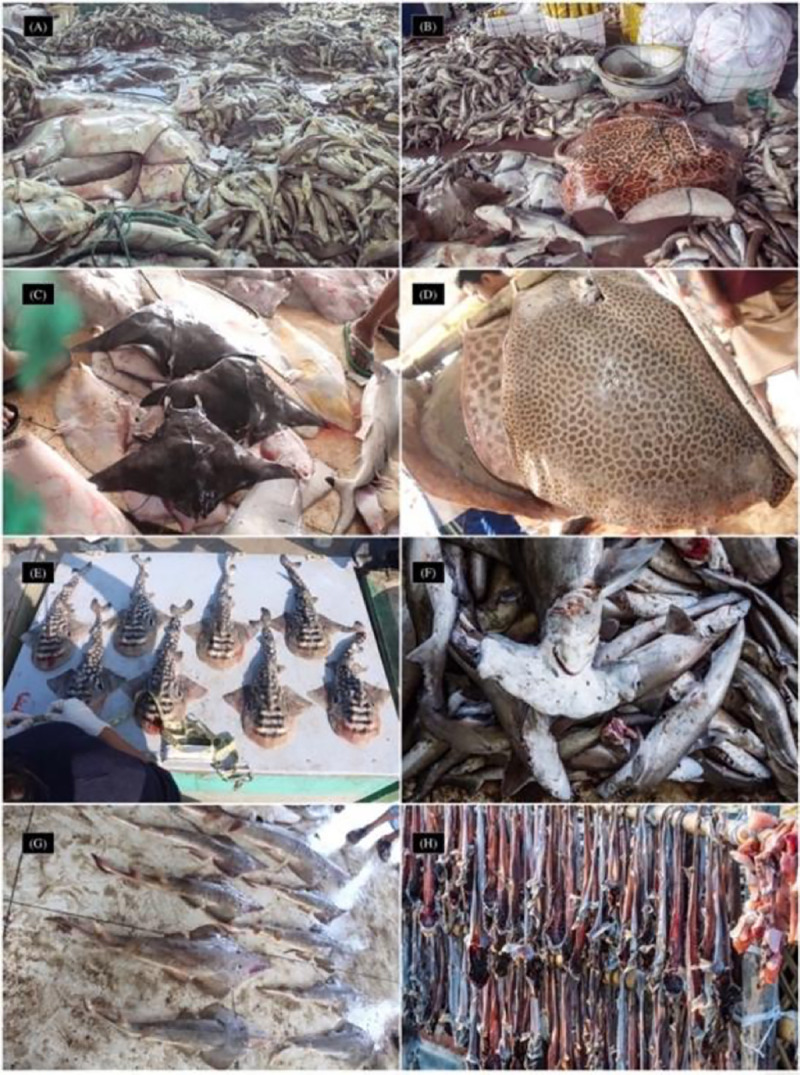
Species at landing site surveys. The piles of elasmobranchs in Cox’s Bazar (A-B) include more than 3000 individuals; (C) Assorted landing of rays (*Mobula* spp. *Gymnura* sp.*)*; (D) *Himantura* spp. and *Maculabatis* sp.; (E) Neonates of *Rhina ancylostoma*; (F) *Sphyrna lewini* and *Scoliodon laticaudus*; (G) *Glaucostegus granulatus* and *G*. *obtusus* and (H) Dried smaller sharks at a processing centre in Cox’s Bazar.

Landing site surveys (Fig 2) were made between 7 am and 2 pm when all landed species were either locally bought or packaged and sent to the processing centres. Here, a particular corner of the landing area was designated for elasmobranch landing and trade. On several occasions, a substantial number of sharks were landed at night, and the data was collected when possible. In Chattogram the survey was conducted in four exclusive shark processing centres, as no designated landing area was found, and all elasmobranch catches were brought to these centres after being purchased in auctions.

A total of twelve processing centres in Chattogram, Cox’s Bazar, Teknaf and St. Martin’s Island were visited to collect information on any additional landings and/or landings that were transported to these centres from the south-central or south-west regions. Traders and workers in the centres were asked to differentiate amongst the landings to avoid double counting any specimens. Although the presence of largetooth and green sawfish has been presented and discussed in other studies [27, 90], this record has been incorporated here for a complete understanding of species composition in artisanal catch and diversity.

A permit was granted by the Department of Forest to study the landed elasmobranchs in the different landing sites and for the collection of DNA samples. Permission was taken to sample specimens from private elasmobranch processing centres from the owners. No specific permissions were required for these locations/activities as the data were collected from carcasses of by-caught fishes, and no harm could have been done. However, in the study, several endangered and protected species were sampled; however, the permit was granted by the Department of Forest to do so. No permit was needed from any Institutional Animal Care and Use Committee or equivalent animal ethics committee as only fishes already dead were sampled, and the method of sacrifice was not applicable. All sampling procedures were reviewed by the Department of Forest and approved.

### 2.5. Additional data on seasonality, distribution, gear used and trade

Information on the landing dates (i.e. season), distribution of landing (i.e. where it was landed), and gear used to catch the particular elasmobranchs was documented where possible. To understand the relationship between species total length (TL), gear mesh size, and seasonality, multiple one-way ANOVA tests comparing TL ~ season, and TL ~ gear were performed. The aim being to recommend potential measures such as gear modification or temporal conservation measures (e.g. fishing bans or quota for a certain season).

Further analysis was also performed to evaluate which gear was catching more elasmobranchs than others, and to the species-specific level where the data was available. One-way ANOVA tests were performed to estimate how different quantitative dependent variables (i.e., total number of specimens landed and the total length of landed specimens) changed following the different levels of categorical variables or factors (i.e., season, gear and species). Graphical checks of the assumption of the models were carried out for constancy of variance, normality of errors and homoscedasticity using the *plot(aov(model))* function in R (R Core team, 2020). To investigate the effects of the different factor levels, the *summary*.*lm* function was used. For evaluating the effect size of the ANOVA model, Eta Squared, Omega Squared and Cohen’s F measure were calculated using *anova_stats(model)* function. Finally, a Tukey’s Honestly Significant Difference (Tukey’s HSD) post-hoc test for pairwise comparisons was performed. For all data analysis and visualisation preparation, R (R Core team, 2020) was used.

Species-specific buyers, price and demand, were observed and documented at landing sites. Market staff and fishers were interviewed regarding gear used to catch the landed species, of which detailed data was collected. Additionally, prices of whole-bodied sharks and shark products, together with information about the role of the buyers (consumers *vs* traders) was collected daily using a simple datasheet.

### 2.6. Relative aggregate landing analysis

Elasmobranch landing data from Sea Around Us (http://www.seaaroundus.org/data/) was downloaded and analysed to compare Bangladesh’s landing data with other Bay of Bengal countries and to evaluate Bangladesh’s contribution to the elasmobranch fishery in this region.

## 3. Results

### 3.1. Annotated checklist

Elasmobranchs were recorded from within almost all ecosystem and habitat types of the Bay of Bengal (S1 Fig). A total of 161 records of elasmobranchs (66 sharks and 95 rays) were identified in the literature as being present or possibly present (ones not recorded yet, but the Bay of Bengal, Bangladesh region is a range) in Bangladeshi waters (S1 Table). Of these, 151 (95.5%) were persistent with correct taxonomy (with updated taxonomy for some) and geographic distribution. One record was questionable, *Hypoprion palasorrah* a shark species reported by Hussain et al., 1970 (could not be corroborated by any present taxa). Fourteen species were likely either wrongly identified, as their geographic ranges do not extend to the Bay of Bengal, or are now synonymous with a different species, e.g. Lesser devil ray/ Atlantic devil ray *Mobula hypostoma* (range is Western Atlantic), the Brown numbfish/ Brown electric ray *Narcine brunnea* (synonumous with *N*. *timlei*), the Smalltooth sawfish *Pristis pectinata* (range is in the Atlantic), the Whitespotted wedgefish *Rhynchobatus djiddensis* (restricted to the Red Sea and the tropical western Indian Ocean to South Africa), the Cownose ray *Rhinoptera bonasus* (restricted to Western Atlantic). Two potential undescribed species were recorded. At least 35 species (21.7%) require further confirmation (S2 Fig) with photographic, genetic or other forms of taxonomic reports (i.e. digital or museum voucher specimens or catalogues).

Moreover, the presence of the remainder was confirmed with recent records, accurate references and personal communications or photographic and genetic evidence. Excluding species with uncertain status and undescribed species, the valid species from Bangladeshi waters total 111 and taxonomic work on an additional few species is underway. This includes more than 18 additional species that have been recently reported. However, it is assumed that the list is still incomplete and needs further taxonomic work for several families.

### 3.2. Surveys: Species composition at landing sites and processing centres

#### 3.2.1. General findings

**Fig 3:**
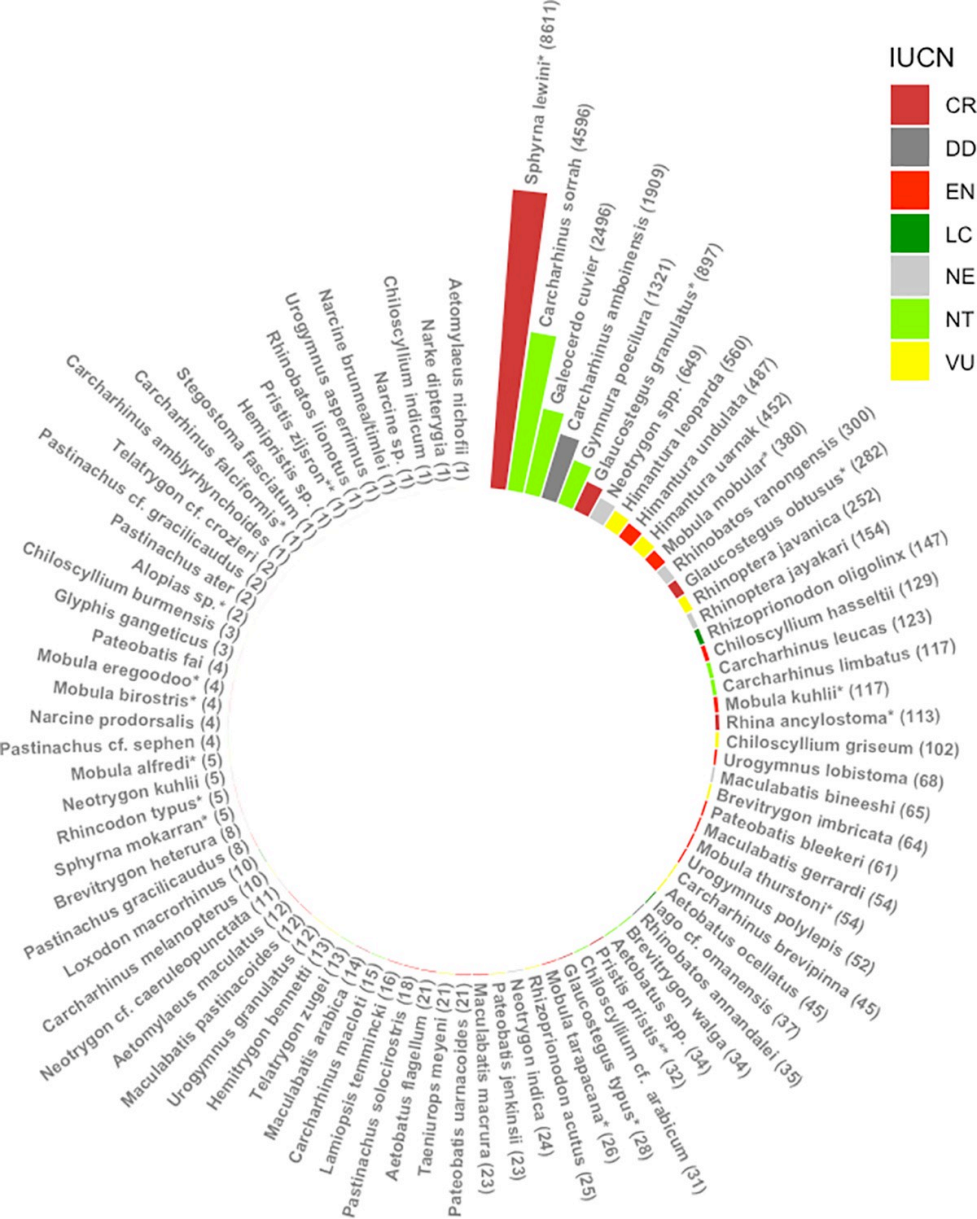
Elasmobranchs documented from the landing sites. Species composition of total elasmobranchs for both sharks and rays recorded by the authors from Cox’s Bazar, Chattogram and St. Martin’s Island during the study period. Species are listed and grouped according to frequency (highest to lowest). *Scoliodon laticaudus* (n = 26280) was not added to this figure for ensuring better visual for the graph. IUCN Red List status is given for each species, or a higher taxon, and colour coded. Here, LC—Least Concern (bottle green), NT—Near Threatened (light green), VU–Vulnerable (yellow), EN- Endangered (red), DD- Data Deficient (grey), NE—Not Evaluated (light grey). Species with ** means listed in CITES app. I and * means listed in CITES CITES App. II.

A total of 162198 individual elasmobranchs were counted. These belonged to 88 species (30 species of sharks, ten species of Rhinopristiformes rays and 48 species of other rays). The total number of species documented were approx. 77.3% of all species present in Bangladesh (Fig 3) belonging to 20 families (eight families of shark and 12 families of rays; seven species need further taxonomic confirmation).

Among all the elasmobranchs that were counted, 94.24% (n = 152849) were sharks, and 5.76% (n = 9349) were rays since rays were more challenging to identify as a result of being landed on their ventral side except for Rhinopristiformes rays which were landed on their dorsal side making it easier to identify. Almost 29.26% of sharks were identified to species level as piles of smaller individuals were virtually impossible to identify in the landing sites. Additionally, 55.57% Rhinopristiformes rays and 82.8% other rays were identified to species level (Table 1). The number of rays was lower than sharks as data on rays were collected when they are landed on their dorsal side hence identifiable, and therefore does not reflect relative abundance. A total of 1120 individuals belonging to 28 species of sharks and Rhinopristiformes rays were sampled for detailed biological and morphometric information (Table 2).

**Table 1:** List of all shark and ray species recorded between January 2016 and December 2019, Global IUCN Red List of Threatened status (EN: Endangered; NT: Near Threatened; VU: Vulnerable; DD: Data Deficient; LC: Least Concern); NE: Not evaluated), assessment dates, CITES, CMS and National protection status are given with commented on their identifications.

Family	Scientific name	Common name	Local name (Bangla)	Number	Notes	CITES	IUCN (Year of last assessment)	National protection	CMS
**Sharks**									
Carcharhinidae	*Scoliodon laticaudus* and *Scoliodon macrorhynchos*	Spadenose Shark and New Spadenose Shar	Churi hangor, Kala hangor	26280	Gravid females with embryos (embryos, n = 11; TL = 5.08 cm in one individual) in January. Fully grown pups upon dissention in April (5–9 pups, TL = 12.7 cm), gravid female TL = 45.72–53.34 cm *S*. *macrorhynchos* was relatively uncommon.	Not listed	NT (2005)	Schedule I (*Scoliodon laticaudus)*	Not listed
Carcharhinidae	*Carcharhinus sorrah*	Spottail Shark	Lota hangor	4596	40 gravid females in March and April (TL = 106.68–158.5; mean = 128.12±20.16). Two dissected, 5 pups each, all female (TL = 45.2–76.2; mean = 57.9±10.54)	Not listed	NT (2007)	Schedule I	Not listed
Carcharhinidae	*Lamiopsis temmincki*	Broadfin Shark	-	16		Not listed	EN (2008)	Not protected	Not listed
Carcharhinidae	*Galeocerdo cuvier*	Tiger Shark	Bagha hangor	2496		Not listed	NT (2018)	Schedule I	Not listed
Carcharhinidae	*Carcharhinus amboinensis*	Pigeye Shark	Bhota/Moilla/Mohila/Goh/ Gundum/ Gongi / Boli hangor	1909	18 gravid females between February and April. Upon dissection (n = 3), 16–17 pups (8 f and 8 m in one; 10 f and 7 m in another)	Not listed	DD (2005)	Not protected	Not listed
Carcharhinidae	*Carcharhinus leucas*	Bull Shark	Bhota hangor	123	One gravid female in April	Not listed	NT (2005)	Not protected	Not listed
Carcharhinidae	*Carcharhinus melanopterus*	Blacktip reef shark	Illissha boli hangor	10		Not listed	VU (2020)	Not protected	Not listed
Carcharhinidae	*Rhizoprionodon acutus*	Milk Shark	-	25		Not listed	VU (2020)	Schedule I	Not listed
Carcharhinidae	*Rhizoprionodon oligolinx*	Grey Sharpnose Shark	Shonali hangor/shonali lota	147	31 gravid females (20 in March and April and 11 in January, TL = 60.96–71.12; mean = 68.07±5.8). 5 dissected, (2–8 pups, mostly all females, in one 2f and 2 m; TL = 17.78–27.94; mean = 23.24±6.18)	Not listed	LC (2003)	Schedule I	Not listed
Carcharhinidae	*Carcharhinus limbatus*	Blacktip Shark	Lota boli hangor/bhota hangor	117	6 gravid females in March and April (TL = 143–152.2)	Not listed	NT (2005)	Schedule I	Not listed
Carcharhinidae	*Carcharhinus brevipinna*	Spinner Shark	Athailla/illissha boli hangor	45		Not listed	VU (2020)	Not protected	Not listed
Carcharhinidae	*Glyphis gangeticus*	Ganges Shark	Bhota/Illissha hangor	3		Not listed	CR (2007)	Schedule I	Not listed
Carcharhinidae	*Carcharhinus amblyrhynchoides*	Graceful shark	-	1		Not listed	NT (2005)	Not protected	Not listed
Carcharhinidae	*Carcharhinus falciformis*	Silky shark	Lota hangor	1		App. II	VU (2017)	Schedule I	App. II
Carcharhinidae	*Carcharhinus macloti*	Hardnose Shark	-	15		Not listed	NT (2003)	Schedule I	Not listed
Sphyrnidae	*Sphyrna mokarran*	Great Hammerhead shark	Haturi hangor/Kaunna	3	All adults and found in winter, further photographic evidence neeeded	App. II	CR (2018)	Schedule I	App. II
Sphyrnidae	*Sphyrna lewini*	Scalloped Hammerhead Shark	Haturi hangor/Kaunna	8611	46 gravid females between March and May (TL = 198.12–372; mean = 280.25±55.33), upon dissection pup number 13–17 (in one 11f and 6 m)	App. II	CR (2018)	Schedule I	App. II
Rhincodontidae	*Rhincodon typus*	Whale Shark	Timi hangor	5		App. II	EN (2016)	Schedule I	App. I & II
Alopiidae	*Alopias* sp.	Thresher Shark	-	2		App. II	VU (2018)	Not protected	App. II
Stegostomatidae	*Stegostoma fasciatum*	Zebra shark	-	1		Not listed	EN (2015)	Schedule I	Not listed
Triakidae	*Iago* cf. *omanensis*	Bigeye Houndshark	-	37		Not listed	LC (2008)	Not protected	Not listed
Hemiscyllidae	*Chiloscyllium hasseltii*	Hasselt’s bambooshark	Bashpata hangor/Bash hangor/Hanno/Bang	129		Not listed	EN (2020)	Not protected	Not listed
Hemiscyllidae	*Chiloscyllium burmensis*	Burmese Bambooshark		3		Not listed	VU (2020)	Not protected	Not listed
Hemiscyllidae	*Chiloscyllium griseum*	Grey Bamboo Shark		102		Not listed	VU (2020)	Schedule I	Not listed
Hemiscyllidae	*Chiloscyllium* cf. *arabicum*	Arabian carpetshark		31	Needs genetic analysis to distinguish species	Not listed	NT (2017)	Not protected	Not listed
Hemigaleidae	*Hemipristis* sp.	Snaggletooth Shark		3	Presumably *H*. *elongata*. Need further specimen collection.	Not listed	VU (2015)	Not protected	Not listed
**Rhinopristiformes rays**									
Pristidae	*Pristis pristis*	Largetooth sawfish	Khotok/Khorkhor/ Aissha/Korat mach	32	One gravid female in April, 5 pups.	App. I	CR (2013)	Schedule I	App. I & II
Pristidae	*Pristis zijsron*	Green sawfish	Khotok/Khorkhor/ Aissha/Korat mach	1		App. I	CR (2012)	Schedule I	App. I & II
Rhinobatidae	*Rhinobatos annandalei*	Bengal Guitarfish	Pitambori/Gerenja	35		Not listed	DD (2008)	Not protected	Not listed
Rhinobatidae	*Rhinobatos lionotus*	Smoothback guitarfish	Pitambori/Gerenja	1		Not listed	DD (2008)	Not protected	Not listed
Rhinobatidae	*Rhinobatos ranongensis*	Ranong guitarfish	Pitambori/Gerenja	300+	Mostly juveniles and adults in bulk landing	Not listed	NE	Not protected	Not listed
Glaucostegidae	*Glaucostegus granulatus*	Sharpnose Guitarfish	Pitambori/Gerenja/ Nangla	897	150 gravid females with embryos (egg cases consisting of 40–60 eggs) in April. A mix of both *G*. *granulatus* and *G*. cf. *granulatus*	App. II	CR (2018)	Schedule I	Not listed
Glaucostegidae	*Glaucostegus* cf. *granulatus*	Sharpnose Guitarfish	Pitambori/Gerenja/ Nangla	-	A slightly morphologically different specimen of *Glaucostegus* was encountered and reported as *Glaucostegus* cf. *granulatus*	-	-	-	-
Glaucostegidae	*Glaucostegus obtusus*	Widenose Guitarfish	Pitambori/Gerenja/ Nangla	282	1 gravid female, 3 pups (2f and 1 m)	App. II	CR (2018)	Not protected	Not listed
Glaucostegidae	*Glaucostegus typus*	Giant Shovelnose Ray	Pitambori/Gerenja/ Nangla	28	Rarely sighted, a few times recorded in piles	App. II	CR (2018)	Not protected	Not listed
Rhinidae	*Rhina ancylostoma*	Bowmouth Guitarfish)	Bang hangor	113	3 gravid females in January (TL = 182.9 cm), 8 pups, all female (TL = 50.5–52.3; mean = 51.5±0.64)	App. II	CR (2018)	Not protected	Not listed
**Rays**									
Dasyatidae	*Urogymnus granulatus*	Mangrove whipray	-	12		Not listed	VU (2015)	Not protected	Not listed
Dasyatidae	*Urogymnus polylepis*	Giant freshwater whipray	-	52		Not listed	EN (2016)	Not protected	Not listed
Dasyatidae	*Urogymnus lobistoma*	Tubemouth Whipray		68	1 gravid female (5 pups, 4f and 1m) in January.	Not listed	EN (2020)	Not protected	Not listed
	*Urogymnus asperrimus*	Porcupine Ray		1		Not listed	VU (2015)	Not protected	Not listed
Dasyatidae	*Maculabatis bineeshi*	Short-tail whipray	Shaplapata	65	Several specimens were reported as *M*. cf. *bineeshi* (n = 7)	Not listed	NE	Not protected	Not listed
Dasyatidae	*Maculabatis gerrardi*	Whitespotted Whipray	Fut shaplapata	54		Not listed	EN (2020)	Not protected	Not listed
Dasyatidae	*Maculabatis arabica*	Pakistan/ Arabic whipray	-	14	Better photographic evidences needed. Several speciemens were designated as *M*. cf. *arabica*	Not listed	CR (2017)	Not protected	Not listed
Dasyatidae	*Maculabatis pastinacoides*	Round whip ray	-	12	Several specimens were reported as *M*. cf. *pastanicoides* (n = 5)	Not listed	EN (2020)	Not protected	Not listed
Dasyatidae	*Pastinachus ater*	Broad cowtail ray	-	2	Due to the absence of the tail at the time of sampling, two specimens were reported as *Pastinachus* cf. *ater* (n = 2)	Not listed	LC (2015)	Not protected	Not listed
Dasyatidae	*Pastinachus* cf. *gracilicaudus*	Narrow cowtail ray	-	2	Differences were found between the NADH2 sequences of the Bangladesh as compared to Borneo specimens. This species is tentatively referred to as *Pastinachus* cf. *gracilicaudus*	Not listed	EN (2020)	Not protected	Not listed
Dasyatidae	*Pastinachus gracilicaudus*	Narrow cowtail ray		8	Morphologically identified.	Not listed	EN (2020)	Not protected	Not listed
Dasyatidae	*Pastinachus *cf.* sephen*	Cowtail ray		4	Though a recent taxonomic study found that *P*. *sephen* is only found in the Western Indian Ocean (Red Sea to Pakistan) (Last & Manjaji-Matsumoto 2010). However, four specimens were morphologically very close to *P*. *sephen* and referred to as *P*. cf. *sephen*	Not listed	NT (2017)	Not protected	Not listed
Dasyatidae	*Pastinachus solocirostris*	Roughnose cowtail ray		18		Not listed	EN (2020)	Not protected	Not listed
Dasyatidae	*Brevitrygon imbricata*	Bengal whipray	-	64	Several specimens were reported as *Bevritrygon* cf *imbricata*	Not listed	VU (2020)	Not protected	Not listed
Dasyatidae	*Brevitrygon walga*	Scaly whipray	-	34		Not listed	NT (2017)	Not protected	Not listed
Dasyatidae	*Brevitrygon heterura*	Dwarf whipray		8		Not listed	NE	Not protected	Not listed
Dasyatidae	*Himantura leoparda*	Leopard whipray	Bagha shaplapata	560		Not listed	VU (2015)	Not protected	Not listed
Dasyatidae	*Himantura uarnak*	Coach Whipray	Bagha shaplapata	452	1 gravid female in April. Fine spotted variants were also reported however, as *H*. *tutul* is not a valid species, they are designated as *H*. *uarnak*.	Not listed	VU (2015)	Not protected	Not listed
Dasyatidae	*Himantura undulata*	Honeycomb whipray	Bagha shaplapata	487		Not listed	EN (2020)	Not protected	Not listed
Dasyatidae	*Pateobatis jenkinsii*	Jenkins’ whipray	-	23		Not listed	VU (2015)	Not protected	Not listed
Dasyatidae	*Pateobatis uarnacoides*	Whitenose whipray	-	21		Not listed	EN (2020)	Not protected	Not listed
Dasyatidae	*Pateobatis bleekeri*	Bleeker’s whipray	-	61	Several specimens were reported as *Pateobatis *cf.* bleekeri*	Not listed	EN (2020)	Not protected	Not listed
Dasyatidae	*Taeniurops meyeni*	Round ribbontail ray	-	21		Not listed	VU (2015)	Not protected	Not listed
Dasyatidae	*Neotrygon* cf. *caeruleopunctata*	Bluespotted maskray	-	11		Not listed	NE	Not protected	Not listed
Dasyatidae	*Neotrygon indica*	Indian Ocean blue-spotted maskray	-	24		Not listed	NE	Not protected	Not listed
Dasyatidae	*Neotrygon kuhlii*	Blue-spotted stingray	-	5		Not listed	DD (2017)	Schedule II	Not listed
Dasyatidae	*Neotrygon* spp.	Mask rays (Bay of Bengal variants)	Nil fut shaplapata	649	19 gravid females (1–2 pups, 1m, 1f), couldn’t identified to species level. Consisting of *N*. *caeruleopunctata*, *N*. *kuhlii or N*. *indica*.	Not listed	NE	Not protected	Not listed
Dasyatidae	*Hemitrygon bennetti*	Bennett’s stingray		13		Not listed	VU (2020)	Not protected	Not listed
Narcinidae	*Narcine prodorsalis*	Tonkin numbfish	-	4		Not listed	DD (2007)	Not protected	Not listed
Narcinidae	*Narcine brunnea/timlei*	Brown numbfish	-	1		Not listed	DD (2007)	Not protected	Not listed
Narcinidae	*Narcine* sp.	Andaman numbfish	-	1	Potential undescribed spices.	Not listed	NE	Not protected	Not listed
Gymnuridae	*Gymnura poecilura*	Long-tailed butterfly ray	Podoni/Projapoti	1321	130 gravid females, 33 sampled between November and April (DW = 61–86.36). Pup number 4 (2f and 2 m, DW = 12.5–13 cm).	Not listed	NT (2006)	Schedule II	Not listed
Mobulidae	*Mobula kuhlii*	Shortfin Devil Ray	Shing Chowain/Badura	117		App. II	EN (2020)	Not protected	App. I & II
Mobulidae	*Mobula mobular*	Giant Devil Ray	Shing Chowain/Badura	380	5 gravid females in April (1 f pup, DW = 91.44 cm)	App. II	EN (2018)	Not protected	App. I & II
Mobulidae	*Mobula birostris*	Giant Manta Ray	Shing Chowain/Badura	4	1 gravid female in April (1 f pup)	App. II	EN (2019)	Not protected	App. I & II
Mobulidae	*Mobula eregoodoo*	Longhorned Pygmy Devil Ray	Shing Chowain/Badura	4		App. II	EN (2020)	Not protected	App. I & II
Mobulidae	*Mobula tarapacana*	Sicklefin Devil Ray	Shing Chowain/Badura	26		App. II	EN (2018)	Not protected	App. I & II
Mobulidae	*Mobula thurstoni*	Bentfin Devil Ray	Shing Chowain/Badura	54		App. II	EN (2018)	Not protected	App. I & II
Aetobatidae	*Aetobatus ocellatus*	Spotted eagle ray	-	45	5 gravid females (one pup each, pup’s DW = 30.5 cm). Several specimens were reported as *Aetobatus* cf. *ocellatus*	Not listed	VU (2015)	Not protected	Not listed
Aetobatidae	*Aetobatus flagellum*	Longhead Eagle Ray	-	21		Not listed	EN (2006)	Not protected	Not listed
Aetobatidae	*Aetobatus* spp.	Whitespotted Eagle Ray	-	34	Comprising of *A*. *narinari* and *A*. *ocelletus*. Further field studies are need to determine appropriate characteristics to distinguish species	Not listed	NT (2006)	Schedule II	Not listed
Myliobatidae	*Aetomylaeus maculatus*	Mottled eagle ray	-	12		Not listed	EN (2020)	Not protected	Not listed
Rhinopteridae	*Rhinoptera javanica*	Javanese Cownose Ray	Chowain	252	3 gravid females in January and April	Not listed	VU (2006)	Not protected	Not listed
Rhinopteridae	*Rhinoptera jayakari*	Oman cownose ray	Chowain	154		Not listed	NE	Not protected	Not listed
**Species needing further photographic and genetic evidences**									
Carcharhinidae	*Loxodon macrorhinus*	Sliteye shark	-	10	Rare in comparison to *Scoliodon laticaudus*. Difficult to identify in piles on smaller sharks	Not listed	LC (2003)	Not protected	Not listed
Hemiscyllidae	*Chiloscyllium indicum*	Ridgebacked Bamboo Shark		1	No photographic evidence could have been collected	Not listed	VU (2020)	Not protected	Not listed
Hemiscyllidae	*Chiloscyllium punctatum*	Brownbanded bamboo shark		1	A juvenile was encountered, however, needs further genetic work to separate from juveniles of *C*. *griseum*		NT (2015)		
Dasyatidae	*Maculabatis macrura*	Sharpnose whisray	-	23		Not listed	EN (2020)	Not protected	Not listed
Dasyatidae	*Telatrygon zugei*	Pale-edged stingray	-	13	Need better taxonomic work.	Not listed	NT (2016)	Not protected	Not listed
Dasyatidae	*Telatrygon* cf. *crozieri*	Indian sharpnose ray	-	2		Not listed	NE	Not protected	Not listed
Narkidae	*Narke dipterygia*	Numbray	-	1		Not listed	DD (2007)	Not protected	Not listed
Dasyatidae	*Pateobatis fai*	Pink whipray		4		Not listed	VU (2015)	Not protected	Not listed
Mobulidae	*Mobula alfredi*	Alfred manta	Shing Chowain/Badura	5	Needs further genetic identification as whole specimens were not encountered	App. II	VU (2018)	Not protected	App. I & II
Myliobatidae	*Aetomylaeus nichofii*	Banded eagle ray	-	1		Not listed	VU (2015)	Schedule II	Not listed
**Unidentified individuals to the species level**									
Sharks									
	*Aetobatus* sp.			>243	1 gravid female in January; Couldn’t identified to species level, Consisting of *A*. *ocellatus or A*. *narinari*. It is difficult to confirm identification for *A*. *ocellatus* or *A*. *narinari* without genetic sampling. Most of these specimens were landed ventrally and sometimes in piles precluding the possibility to individually sample them.				
	*Mobula* sp.			>243	Smaller specimens. For larger specimens, they mostly landed ventrally and also in a busy landing sites it was only possible to count without sampling each specimen.				
	*Maculabatis* sp.			>324	Lack of standardised photos and the difficulties in differentiating *M*. *macrura*, *M*. *gerrardi* and *M*. *arabica* in large piles preclude the authors for species-specific identification.				
	*Pateobatis* sp.			>265	3 gravid females between February and April. Species-specific identification was not possible due to ventral landing, in many cases absence of tails and inability of individual sampling in the crowded landing sites				
	*Glaucostegus* sp.*/ Rhinobatos* sp.			>1350	Comprising of *G*. *typus*, *G*. *granulatus and R*. *ranongensis*, *R*. *lionotus*, *R*. *annndalei*> Ventral landing in piles of hundreds of specimens precluded species-specific identification.				
Gymnuridae	*Gymnura* sp.	Butterfly ray		11	Absence of tail and ventral landing precluded species-specific identification.				
Hemiscyllidae	*Chiloscyllium* spp.	Bamboo shark		159	Piles of many specimens and absence of standardised photos precluded species-specific identification.				
		Small unidentified requiem sharks		107743	Piles of thousands of specimens landing precluded individual sampling and species-specific identification.				
		Unidentified large requiem shark		225	Absence of standardised photographs, morphologically similar species landing where only ventral side is visible, absence of fins while landing and inability to individually sampling each specimen in a busy and crowded landing site precluded species-specific identification.				

**Table 2:** Total number (n) and percentage of total (%) of species (elasmobranchs) identified to species level and recorded from Chattrogram, Cox’s Bazar and St. Martin’s Island during the study period (January 2017 to June 2017).

Species	n (Cox’s Bazar)	n (Chattogram)	n (St. Martin’s Island)	Total number of individuals	% total of all species combined	Estimated total weight (kg)	n sampled with precision, size range (mean±S.D.cm)
*Scoliodon laticaudus*	22535	3625	120	26280	58.86	~ 8919	n = 262; 15.24–81.44 (38.33±9.82)
*Sphryna lewini*	7404	1192	15	8611	19.29	~ 31041.5	n = 264; 15.24–304.8 (39.67±19.81)
*Carcharhinus sorrah*	4572	23	1	4596	10.29	~ 671.1	n = 53; 24.38–204.22 (73.31±42.83)
*Galeocerdo cuvier*	2440	31	25	2496	5.59	~ 3177	n = 81; 33.53–550 (124.31±60.55)
*Carcharhinus amboinensis*	1812	86	11	1909	4.28	~ 7019.9	n = 117; 33.53–292.61 (116.14±46.94)
*Chiloscyllium spp*.	461	12	22	234	<1	-	n = 46; 43–75.5 (64.47±9.49)
*Carcharhinus limbatus*	41	76	0	117	<1	~695	n = 19; 45.72–259.08 (130.98±56.07)
*Carcharhinus brivipinna*	45	0	0	45	<1	358	n = 28, 76.2–121.92 (102.73±16.15)
*Rhizoprionodon oligolinx*	135	12	0	147	<1	-	n = 17; 61–65.5 (63.43±1.88)
*Carcharhinus leucus*	121	2	0	123	<1	-	n = 33; 91.44–176 (155.23±27.85)
*Iago* **cf.** ***omanensis***	37	0	0	37	<1	13.09	n = 37; 34.30–53.34 (44.95±4.86)
*Glaucostegus granulatus/G*, cf. *granulatus*	619	278	0	897	53.12	~ 4300.80	n = 137; 42.67–213.36 (105.40±32.53)
*Glaucostegus typus*	1	127	0	128	7.58	-	n = 1; 106.68
*Glaucostegus obtusus*	157	25	0	182	10.78	~ 533.2	n = 22; 60.96–137.17 (97.40±21.82)
*Rhina ancylostoma*	101	9	3	113	6.69	~102	n = 13; 198.12–155.45 (168.66±25.56)

#### 3.2.2. Sharks

A total of 152849 individual sharks belonging to 30 species of eight families were counted and recorded between 15 January 2017 and 21 June 2017 at the four landing sites. Of these, 44722 (29.26%) were identified to species level based on morphological characteristics. The most commonly observed shark species in the landings were the Spadenose shark *Scoliodon laticaudus* (n = 26280; 58.85%), followed by the Scalloped hammerhead shark *Sphyrna lewini* (n = 8611; 19.29%). The Spottail shark *Carcharhinus sorrah*, the Tiger shark *Galeocerdo cuvier* and the Pigeye shark C. *amboinensis*, comprised approximately 10.29%, 5.59% and 4.27%, of the total sharks, respectively (Table 1). Bamboo sharks *Chiloscyllium sp*., Blacktip sharks *C*. *limbatus*, Bull sharks *C*. *leucas*, and Grey sharpnose sharks *Rhizoprionodon oligolinx* were also present in lower number. Milk sharks *Rhizoprionodon acutus*, Hardnose shark *C*. *macloti*, Spinner sharks *Carcharhinus brevipinna*, Graceful sharks *Carcharhinus amblyrhynchoides*, Ganges sharks *Glyphis gangeticus*, Broadfin sharks *Lamiopsis temminckii* and the Thresher shark *Alopias* sp. were in low numbers, with each species comprising less than 1% of the total landings.

Occasionally, individuals of *S*. *laticaudus*, *R*. *acutus*, *R*. *oligolinx* and pups of *C*. *sorrah*, *C*. *limbatus*, *C*. *macloti* and several unidentified requiem sharks were landed in piles of up to 10000 individuals. Identification of all individuals within the pile was difficult though a total of 107743 such individuals were labelled as unidentified smaller sharks.

### 3.2.3. Rays

- Rhinopristiformes rays: A total of 1689 individuals of Rhinopristiformes rays, comprising ten different species, were identified. The most commonly caught species was the Sharpnose guitarfish *Glaucostegus granulatus* and *G*. cf. *granulatus* (n = 897, 53.12%) followed by the Ranong guitarfish *Rhinobatos ranongensis* (n = 300+, ~18%) and Widenose Guitarfish *Glaucostegus obtusus (*n = 282, 16.58%), *the* Bowmouth Guitarfish *Rhina ancylostoma* (n = 113, 6.69%), the Bengal Guitarfish *Rhinobatos annandalei* (n = 35, 2.07%) and the Giant Shovelnose Ray *Glaucostegus typus* (n = 28, 1.66%). Thirty-four sawfishes, including two species (the Largetooth sawfish *Pristis Pristis*, the Green sawfish *P*. *zijsron*), were recorded. However, the sawfish records were presented and discussed separately [27, 90].
- Other rays: A total of 5224 individual (from 6310) rays belonging to 48 species of nine families were counted and identified to species level between January 2016 and December 2019 at the four landing sites. The most common rays were whiprays and stingrays (family: Dasyatidae), comprising the highest number of species [24]. This was followed by the family Mobulidae (6 species) and Aetobatidae (3 species). The families of Mylobatidae, Narcinidae, and Rhinopteridae each had two species recorded, and Mylobatidae, Gymnuridae and Narkidae each had one species documented (Fig 3, Table 1).

Although the majority of the species were from the family Dasyatidae, the most commonly observed ray species by the relative number landed was from the family Gymnuridae (the Longtail butterfly ray *Gymnura poecilura*, n = 1321, 26.23%). This was followed by Bluespotted maskray *Neotrygon* spp. (n = 689; 13.68%). The Leopard whipray *Himantura leoparda*, Honeycomb whipray *Himantura undulata*, Reticulate whipray *Himantura uarnak* each contributed approximately 11.12% (n = 560), 9.67% (n = 487) and 8.97% (n = 452), respectively. Within the family Dasyatidae, other common species found were the Short-tail whipray *Maculabatis bineeshi* (n = 65), the Bengal whipray *Brevitrygon imbricata* (n = 64) and the White-spotted whipray *Maculabatis gerrardi* (n = 54), Arabic whipray *Maculabatis acabica* (n = 14) and other whiprays *Pateobatis* spp., and the rest were of minor abundance, with each species comprising less than 1% of the total landings. Coastal and freshwater species dependent on mangroves were also quite frequently found and included the Giant freshwater stingray *Urogymnus polylepis* (n = 52), Tubemouth whipray *U*. *lobistoma* (n = 68) and mangrove whipray *U*. *granulatus* (n = 12), a number of unidentified individuals of the same genus.

Cownose rays (family: Rhinopteridae) were also frequently landed. From these, the most common species found were the Flapnose ray or Javanese cownose ray *Rhinoptera javanica* (n = 252, 5%) and Oman cownose ray *R*. *jayakari* (n = 154, 3.06%). From the family Mobulidae six species (585; 11.61%) were identified to species level based on morphological characteristics. The most commonly observed species in the landings was the Giant devil ray *Mobula mobular* (n = 380), followed by the Shortfin devil ray *M*. *kuhlii* (n = 117), the Bentfin devil ray *M*. *thurstoni (n = 54)* and the Chilean devil ray *M*. *tarapacana* (n = 26). Of the species the Giant oceanic manta ray *M*. *birostris* and the Longhorned pygmy devil ray *M*. *eregoodoo*, each contributed fewer than ten individuals.

Eagle ray landings from two families were commonly recorded (Aetobatidae and Myliobatidae), with five different species identified. The most commonly caught species was the Ocellated eagle ray *Aetobatus ocellatus* (n = 45), followed by the *Aetobatus* cf. *ocellatus (*n = 34), the Longheaded eagle ray *Aetobatus flagellum (*n = 21), the Mottled eagle *Aetomylaeus maculatus* (n = 12) and the Banded eagle ray *Aetomylaeus nichofii* (n = 1) (needing further confirmation). A total of 2425 individuals belonging to the genus *Aetobatus*, *Mobula*, *Maculabatis*, *Pateobatis*, *Glaucostegus* and *Rhinobatos* could not be identified to the species level.

#### 3.2.4. Maturity in recorded species

**Fig 4:**
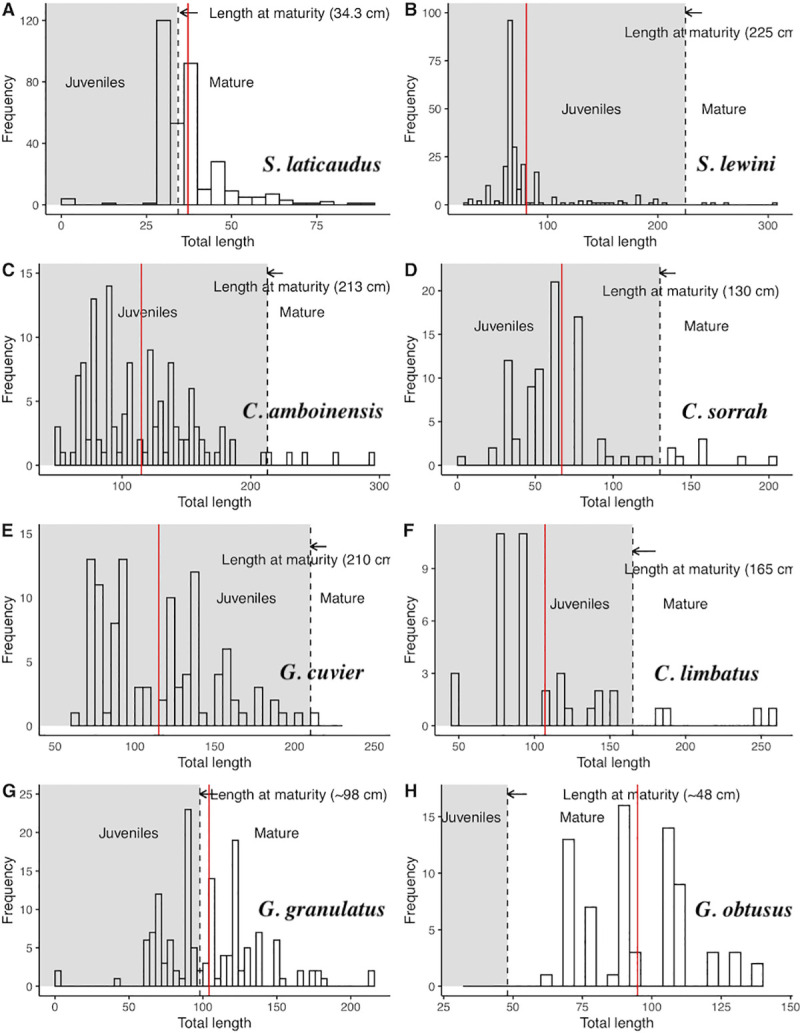
Length frequency with mean (red line), juveniles (shaded grey) and mature specimens of (A) *S*. *laticaudus*, (B*) S*. *lewini* (C) *C*. *amboinensis*, (D) *C*. *sorrah*, (E) *G*. *cuvier*, (F) *C*. *limbatus*, (G) *G*. *granulatus* and (H) *G*. *obtusus*. The dashed line indicates the proportion of juveniles and the red line indicates the mean length of each species.

**Fig 5:**
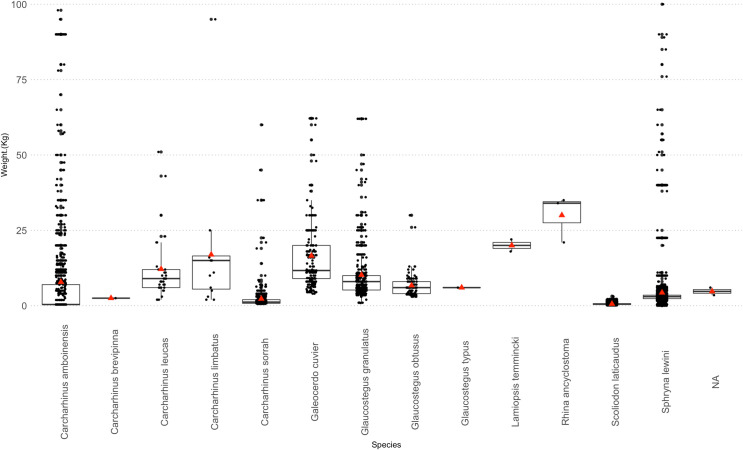
**Range of weights of elasmobranch species landed during the study period with mean (red triangle).** Weights of greater than 100 kg were mostly estimated hence are not shown in the figure. The weight range in kg for sampled specimens were as follows: *C*. *amboinensis* (0.4–450, mean = 9), *C*. *brevipinna* (2.5), *C*. *leucas* (2–51, mean = 12.14), *C*. *limbatus* (2–95, mean = 26.44), *C*. *sorrah* (0.6–60, mean = 2.28), *G*. *cuvier* (4–62.14, mean = 16.54), *G*. *granulatus* (0.9–62, mean = 10.21), *L*. *temmincki* (18–22, mean = 20), *S*. *laticaudus* (0.09–2, mean = 0.604), S. lewini (0.4–200, mean = 4.38).

Based on length at maturity [86, 87] of the sampled specimens for large species, the majority of landed sharks and rays were juveniles (n = 18663 out of 18999 sampled); *C*. *amboinensis* (n = 1481, 99.6%), *C*. *sorrah* (n = 3482, 99.5%), *S*. *lewini* (n = 10107, 99.7%), *C*. *limbatus* (n = 23, 67.65*%)*, *G*. *cuvier* (n = 3225, 100%), *G*. *granulatus* (n = 343, 69.4%). However, no specimen of *G*. *obtusus* (n = 121) sampled was juvenile, whereas <1% (n = 750 out of 32970 sampled) of the individuals of *S*. *laticaudus* was found to be juveniles (Fig 4). Weight varied according to species. The majority of the individuals were less than 25 kg for large specimens; however, for smaller specimens like *S*. *laticaudus* and pups of other species, many individuals were less than one kg (Fig 5).

### 3.3. Insights on seasonality, distribution, gear used and trade

#### A. Seasonality

**Fig 6:**
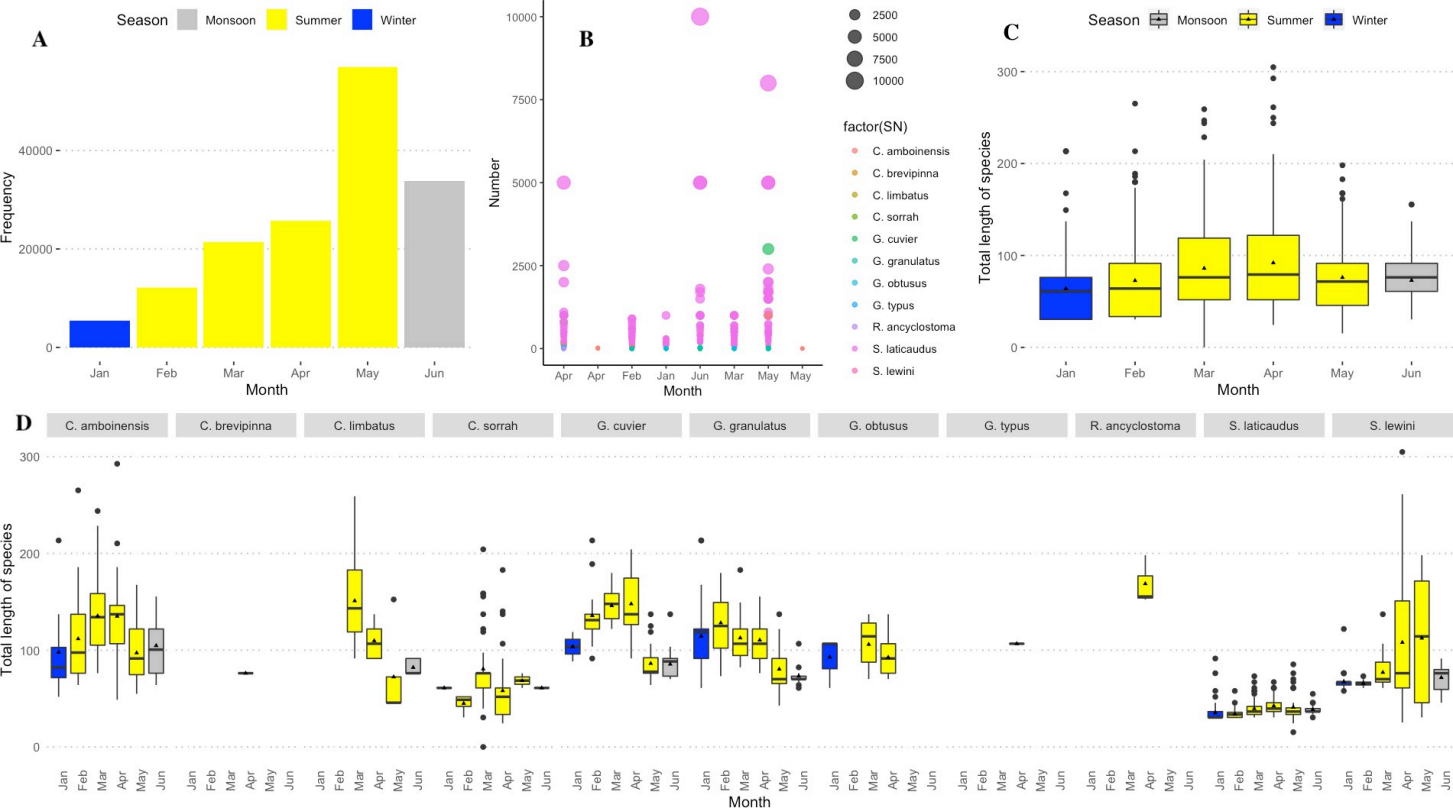
Seasonality: **(A)** Relative frequency of recorded specimens of elasmobranchs per month of the study period, (**B**) species-specific frequency of landing for each month, where the size of the circle denotes the number of bulk landings in a single day, (**C**) overall range of length of landed elasmobranchs in each month, and (**D**) length-specific landing for each species per month of the study period.

There was a significant difference in the number of specimens landed in different months (p< 0.001, F-statistic 9.081 on 7 and 1174 DF, Intercept 95.76, etasq 0.051, partial.etasq 0.051, omegasq 0.046, partial.omegasq 0.46, cohens.f 0.233, power 1: 100% chance of finding a statistically significant difference) and season (p <0.001, F-statistic: 11.68 on 2 and 1179 DF, Intercept 362.95, etasq 0.019, partial.etasq 0.019, omegasq 0.018, partial.omegasq 0.018, cohens.f 0.141, power 0.994: 99% chance of finding a statistically significant difference). Here the effect size of the model is small. The number of sharks landed was substantially higher in the pre-monsoon and monsoon season, followed by summer for both large and small species (Fig 6A). The Tukey’s HSD test showed, there was a significant difference between summer-monsoon (P< 0.004) and winter- monsoon (p<0.0001). However, no significant difference was found between winter and summer regarding the number of landing. The model validation graph showed no large outliers that would cause bias in the model, and the mean of the residuals was horizontal and centered on zero.

However, as detailed data could only be collected for one month during winter (i.e. January), this result shows the frequency mostly from summer to monsoon. The most considerable bulk of smaller species (e.g. *S*. *laticaudus*) were observed in May and June (Fig 6B). The larger specimens were mostly caught in summer and pre-monsoon (Fig 6C and 6D).

#### B. Distribution

**Fig 7:**
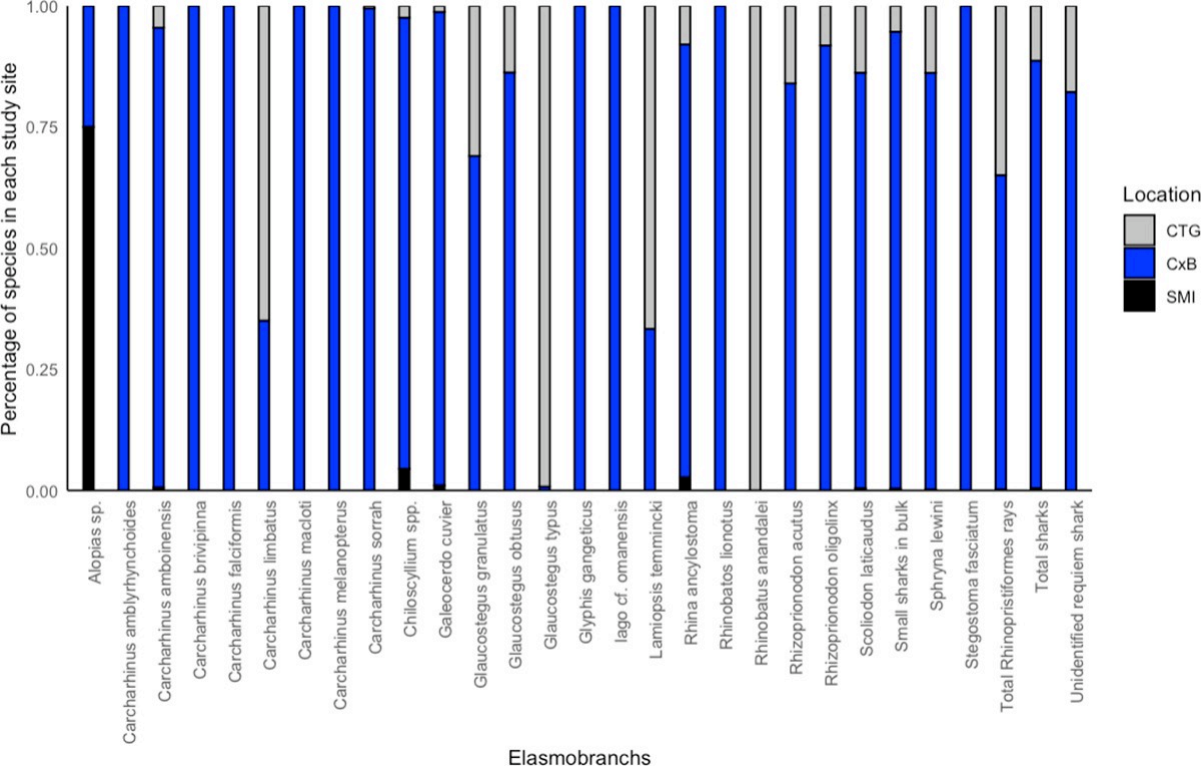
Distribution of sharks studied (at landing sites). Here, CTG = Chattogram, CxB = Cox’s Bazar and SMI = St. Martin’s Island.

The highest number of sharks and rays were landed in Cox’s Bazar, followed by Chattogram, and the lowest in Teknaf followed by St. Martin’s Island (Fig 7).

#### C. Gear

**Fig 8:**
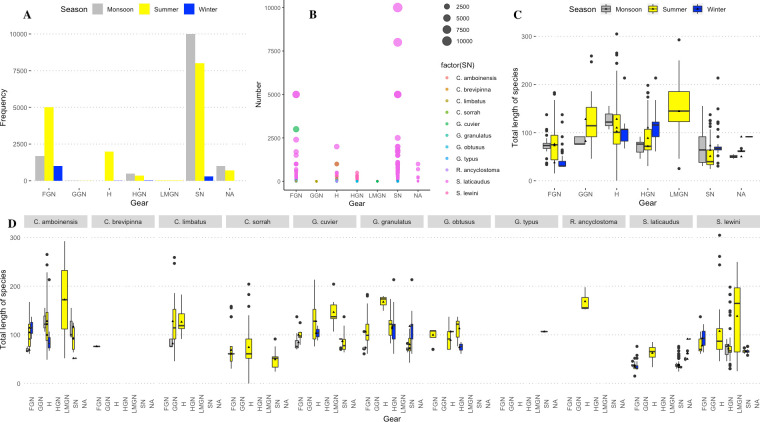
(A) Frequency of species caught in each documented gear type, (B) species-wise bulk landing in each gear type, (C) overall length-specific landing in each gear type, and (D) range of total length of different elasmobranch species and different gear used to catch the reported specimens (species-wise). Here, FGN = floating gillnet, GGN = general gillnet with varying mesh sizes, H = hooks, HGN = hilsa gill net, LMGN = large mesh gillnet (Lakkha jal), and SN = seine net.

Sharks were caught by gillnets (mesh size between ~10 and 32 cm), set bag nets and longline hooks or individual hooks. Less than 1% of the individuals (n = 1387) were caught by using non-baited long lines targeting rays or other smaller fish, or individual bigger iron hooks targeting groupers or any opportunistic big fish. In several cases (n = 21), bigger elasmobranch species (*C*. *amboinensis*, *C*. *leucas*, *G*. *cuvier*, *G*. *granulatus*) were documented while the hooks were still attached to the jaws. Floating drifting gill nets caught 14.9% of the individuals (n = 15175), predominantly targeting Hilsa (*Tenualosa ilisha)*, and 2.71% (n = 2754) were caught using submerged gill nets. Less than 1% (n = 143) were caught in the Lakkha net (mesh size larger than 30 cm), and 66.45% (n = 67084) were caught by either seine net or gillnets targeting different fishes. In 14.92% of cases, the gear used to catch the individuals could not be recorded (Fig 8A).

Floating gill nets and seine nets caught significantly more sharks than any other nets (but mostly smaller specimens in seine nets) (p<0.001, F-statistic: 10.93 on 5 and 1167 DF, Intercept 100.93, etasq 0. 045, partial.etasq 0. 045, omegasq 0.041, partial.omegasq 0.041, cohens.f 0.216, power 1: 100% chance of finding a statistically significant difference) (Fig 8B). Other rays are predominantly caught in targeted non-baited long lines deployed in the shallow water coastal areas. They are also caught in other gears as by-catches. There was a significant positive relationship between gear type (mesh size of the nets used) and the increasing length of the elasmobranchs (Fig 8C). ANOVA models resulted in positive relationships with gears and increasing total length (p<0.001, F-statistic: 99.34 on 5 and 1158 DF, Intercept 63.3, etasq 0.300, partial.etasq 0.300, omegasq 0.297, partial.omegasq 0.297, cohens.f 0.655, power 1: 100% chance of finding a statistically significant difference). The model effect size is moderately large. The positive relationship was found for generally gillnets (p<0.03) both for floating gillnets (p<0.001), large mesh gillnets (p<0.0001), hooks (p<0.0001). For seine nets (p<0.0001) negative relationship was reported. However, no significant relationship was found for particularly hilsa gillnets, probably because they catch all size of sharks in abundance. Species-specific length concerning gears is shown in Fig 8D.

#### D. Trade

86.37% of the species were bought from the landing site by either non-fisher tribal men in the coastal region/ intermediaries/ middlemen to deliver to the processing centres. Tribal or Hindu men bought less than 1% (n = 300) to eat fresh, and in 38 cases, Myanmar citizens were reported to buy the fish directly from the landing site. This information needs further confirmation. In 13.41% of cases, the buyer could not be documented on-site.

### 3.4. Threatened status and protection of species recorded

The majority of the species recorded are threatened according to IUCN Red List (CR = 10, EN = 22 and VU = 22), 12 are NT, seven are Data Deficient, with the remainder Not Evaluated. Amongst all, only 37 species receive some level of global or national protection. Nineteen species are protected under national law: The Wildlife (Conservation and Security) Act, 2012 (Schedule I = 15 species and Schedule II = 4) (Table 1).

**Fig 9:**
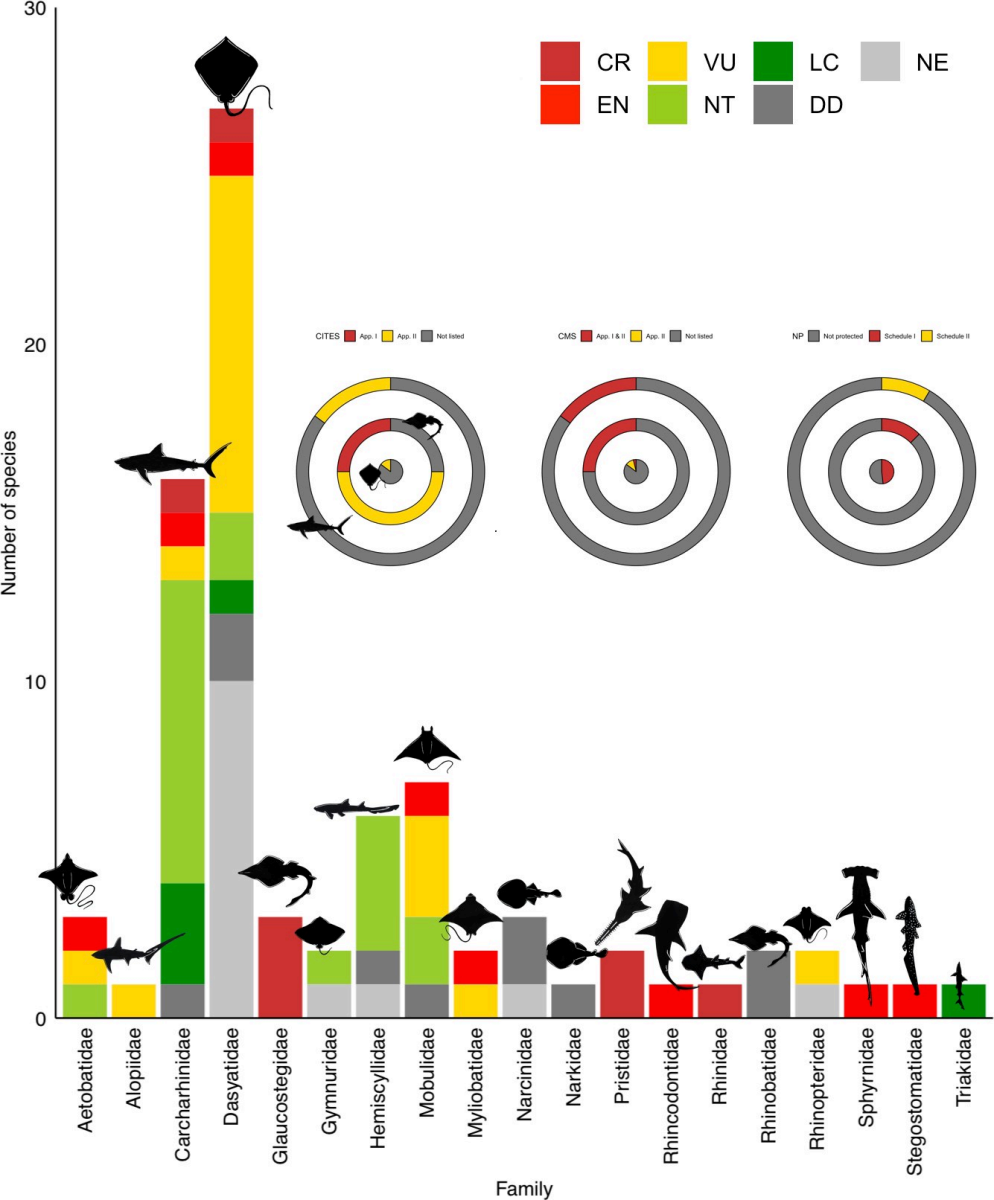
Threatened status of each species within each family recorded in the study. Number of species recorded for each elasmobranch family from artisanal fisheries of south-eastern Bangladesh. IUCN Red List status, CITES App. Listings and CMS listings are also shown in circular graphs, including level of national protection under the Wildlife (Conservation and Security) Act, 2012.

Regarding international trade regulation, 16 species are listed in App. II (Glaucostegus spp., *Mobula* spp., *C*. *falciformis*, *R*. *typus*, *S*. *lewini*) and two are in App. I (sawfishes) of the Convention on International Trade in Endangered Species of Wild Fauna and Flora (CITES). Furthermore, ten species are designated in App. I and II and four in App. II of the Convention on Migratory Species (CMS) (Fig 9).

The highest level of protection is given to sawfishes, *Mobula mobular* (mentioned as *M*. *japanica* in the Wildlife (Conservation and Security) Act, 2012), *C*. *falciformis*, *R*. *typus*, *S*. *lewini* by all three mechanisms (i.e. national law, CITES and CMS). The rest of the *Mobula* spp. are protected by both CITES and CMS. Eleven of the CITES listed App. II species are not protected by the national law, and seven CR, 18 EN and 15 VU species are also not protected by the national law (Table 1).

### 3.5. Relative aggregate landings

**Fig 10:**
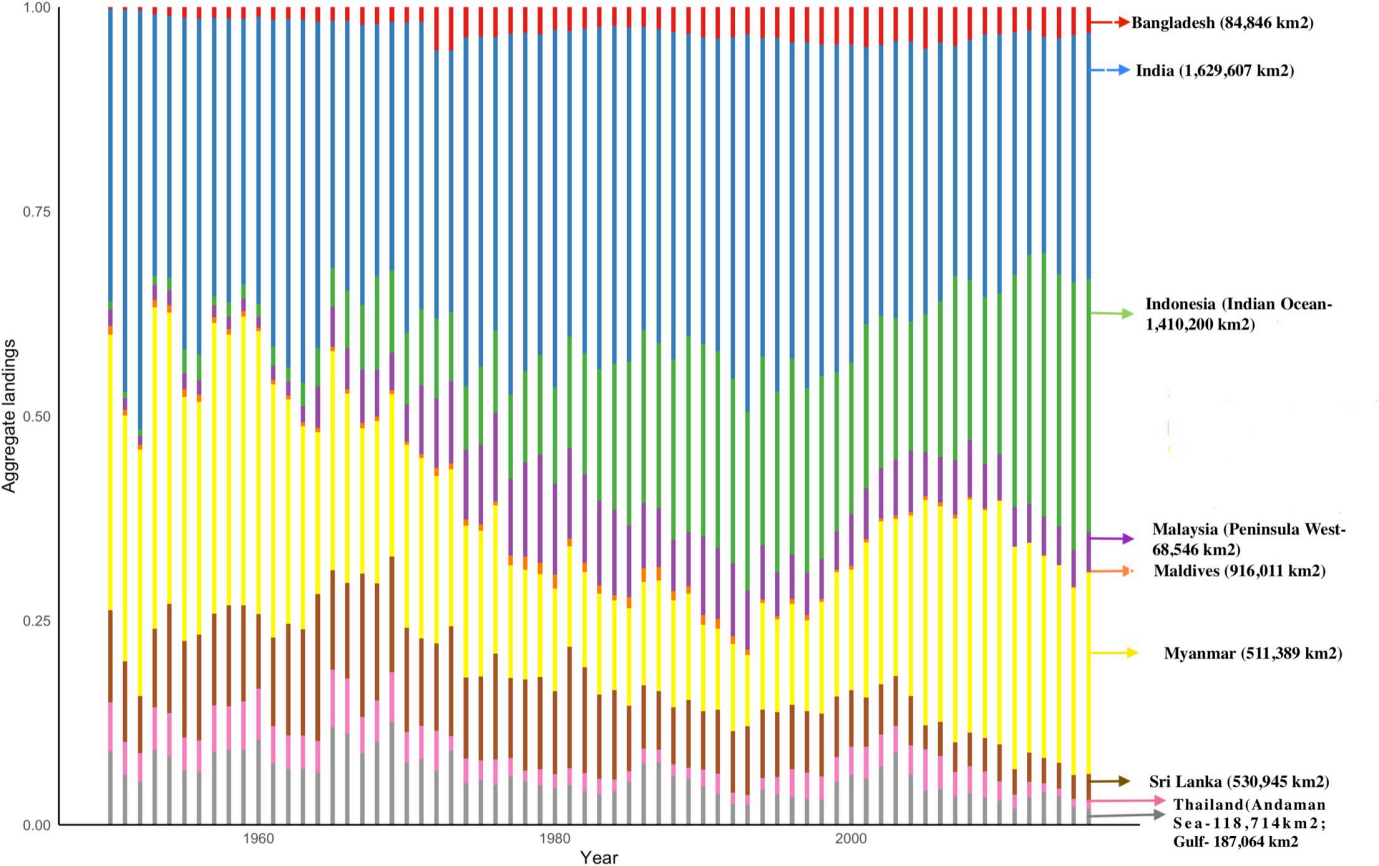
Relative aggregate landings of elasmobranchs in the Bay of Bengal countries from 1950–2016. Data from Sea Around Us: http://www.seaaroundus.org. The area of the EEZ is added for each country at the right side of the graph to illustrate the relative area in which fishing activities are operated.

The average reported landing of aggregate elasmobranchs decreased from 10909 t in 2000 to 7163 t in 2014 and about 6000 t in 2016 [41]. Bangladesh contributes 4% to the elasmobranchs caught from the Bay of Bengal region on an average from 1950–2016 (Fig 10, S3 Fig, S2 Table), utilising relatively smaller Exclusive Economic Zone (EEZ) than the majority of other countries.

## 4. Discussion

Our study found that elasmobranchs are being caught and landed in unmonitored sites in the Bay of Bengal, Bangladesh and that catches include globally threatened and nationally protected species. Elasmobranchs are an essential component of Bangladesh’s marine biodiversity that supports extensive artisanal fisheries. Species composition in the Bangladeshi Bay of Bengal was evaluated to explore elasmobranch diversity and conservation implications. The total diversity of elasmobranchs recorded in this study is substantially higher than reported in previous studies. Elasmobranchs were caught with a variety of fishing gear, both as by-catch and targeted fishery, suggesting a need for catch and landing monitoring. Elasmobranch landings and catch surveys in the neighbouring countries, especially in India and Myanmar, indicate that the Bay of Bengal is a hotspot for several threatened, genetically distinct, and globally important species.

This study provides enhanced knowledge of elasmobranch species composition, relative abundance, seasonality and gear used in the artisanal fishery. An essential initial baseline for encouraging evidence-based decision making in the Bay of Bengal, Bangladesh has been offered. By collaboratively combining our knowledge base to inform ecology, socioeconomics, conservation and trade concerns, a suite of next steps for effective governance and priority research can be initiated to stop the collapse of the most depleted species and promote sustainable approaches for others.

### 4.1. High diversity of elasmobranchs in the Bay of Bengal

Previous estimations of elasmobranch diversity in Bangladeshi waters have been significantly underestimated (35 to 81 in different studies) [34, 35, 71, 74, 77, 80, 83, 84, 86, 91, 92]. By recording artisanal catch, the study has raised the number of reported elasmobranch species in Bangladesh by thirteen. By inclusion of those which were previously misidentified or lacked supporting evidence and incorporating unreported deep-sea species, the report of total diversity has only increased (see annotated checklist). The previous underestimation is likely in part due to poor coverage of migratory species, deep-water species, rare species [93] and discarded individuals in the industrial catch [94]. Additionally, different fishing efforts with vessel and gear characteristics, target species, capacity and exploited diverse habitats within the artisanal fisheries, which were previously studied, may have limited preparing a complete list due to the difficulty at working in the widely distributed informal and disperse landing sites.

Moreover, the lack of exploratory surveys and limited trained workers at the landing sites made it even more cumbersome. The nature of landing recording systems may have hampered accurate elasmobranchs accounts in Bangladesh. They aggregate all species into one group, masking species level exploitation status and relative abundance [69]. Therefore, it is likely that elasmobranch species richness and endemicity have historically been underestimated, suggesting the potential for additional, evolutionary important species to exist in the region [26]. Of rare and endemic elasmobranchs, the Ganges shark has been previously reported after a decade [85] and also found in this study. However, a total of 140 Ganges shark jaws were tracked back to Bangladesh within four years (probably between 2016 and 2019), which suggests insufficient documentation by species-level reports in previous studies [27].

The results have substantial implication in the global context. Bangladesh has proven to be a high biodiverse region regarding elasmobranchs (confirmed 111 species in the annotated checklist) compared to neighbouring countries. Elasmobranch species richness recorded in Bangladeshi waters is higher than that of other Indian Ocean countries and regions including Bay of Bengal such as the Arabian Gulf, which has 43 shark species [95], Sri Lanka (92- >100 elasmobranchs) [96, 97], Maldives (51 elasmobranchs) [98] and Andaman and Nicobar Islands reporting 57 species [99]. Elasmobranch diversity in Bangladesh is also possibly higher than in Thailand (145 elasmobranchs) [100]. Bangladesh possesses similar levels of elasmobranch diversity with India and Indonesia (reporting at least 118(137–207) species) [101–104], although the latter countries have a much larger marine fishing area. Hence, improved taxonomic and conservation studies are urgently needed in Bangladesh.

### 4.2. Relative abundance in artisanal catch

The relative abundance of elasmobranchs was disproportionately higher for small-bodied sharks and rays. This might be explained by the fact that many elasmobranch species use inshore nutrient-rich waters and mangroves as nursery grounds [105]. Carcharhinids were the most abundant sharks reported in this study. The spadenose shark *S*. *laticaudus* was the most commonly documented shark species, likely due to its relatively high fecundity and occurrence in shallow-water (13 m) demersal habitats [86]. As such, spadenose sharks are frequently exploited by large numbers of artisanal boats, which deploy gear in great numbers [50], including the small mesh monofilament gillnet. After spadenose shark, other abundant shark species were spottail shark *C*. *sorrah*, blacktip shark *C*. *limbatus*, tiger shark *G*. *cuiver* and pigeye sharks *C*. *ambionensis*, followed by bull shark *C*. *leucas* with very low landing of *Carcharhinus falciformis*, *C*. *amblyrhynchoides*, *C*. *brevipinna* and *C*. *macloti*. Similarly, whereas the scalloped hammerhead shark was very commonly caught at all landing sites, other hammerhead sharks (e.g. winghead shark *E*. *blochi*, great hammerhead shark, *S*. *mokarran*) [35, 77], were either not recorded or very rarely found. However, they were previously reported as being abundant in this region. This discrepancy may be due to a severe population decline in the region, possibly driven by extremely valuable fin trade [71] (see details in S3 Table).

Deep-sea, pelagic and migratory elasmobranchs are quite unlikely to be caught in abundance in the shallow depths predominantly fished by Bangladeshi artisanal fisheries. For example, whale sharks and thresher sharks were poorly reported, though there were anecdotal whale shark reports in industrial fisheries (news articles, pers. comm. 2019). However, pelagic species may use inshore waters as breeding grounds [18]; hence the abundance of species like the tiger shark, *Galeocerdo cuvier* or scalloped hammerhead sharks, *Sphyrna lewini* while they were pregnant or in their adult stages were common. The case was similar to Devil rays, Cownose rays and Eagle rays. They were comparatively less common as they are pelagic or benthopelagic and may not overlap with the artisanal fisheries.

There is an acute lack of fisheries data and research surveying industrial fisheries. Similarly, this study did not take into account the industrial catches by the bottom and mid-water trawlers that exploit waters of 200m depth and beyond, meaning that the Bay’s deepest waters are still unrepresented. It is, therefore, possible that deep-water elasmobranchs not previously recorded in the Bay of Bengal may be present, such as hound sharks. Whereas *Mustelus mosis*, a deep-water hound shark, was reported for the first time from the southwest coast [84], *Mustelus manazo*, *Mustelus kanekonis/ Mustelus griseus*, *Iago garricki*, *Iago* cf. *omanensis* were also recorded previously but were not commonly encountered at all [34, 35, 106]. Although landing data is not a perfect proxy to understand the abundance of these species at sea [24], these can give us an idea about the impacts of fishing if long term data is unavailable.

Rays from the family Dasyatidae were the most abundant, consistent with previous reports [34, 35, 77, 80, 92], likely because it comprises a large group of rays consisting of 19 genera and 86 species [87], which inhabit an array of habitats and depths. The most commonly found ray was *G*. *poecilura* from this family, potentially due to higher breeding potential than many other elasmobranch species [107]. The number of juveniles of this species encountered was highest in the winter and pre-monsoon season, probably due to overlapping breeding season and fisheries and the presence of inshore nursery grounds [107]. The second most abundant species sampled belonged to the genus *Neotrygon*, which is characterised by reef-associated or demersal inshore species. *Neotrygon* habitat overlaps with artisanal bottom net fisheries and longline hooks, resulting in high numbers of landings, although demand is comparatively low. The shallow bottom habitats of stingrays and whiprays (e.g. *Himantura* spp., *Maculabatis* spp., *Brevitrygon* spp., and *Pateobatis* spp., were found to be heavily exploited. Numbfishes were found to be rare in landings, potentially due to discards and lower market value.

Rhinopristoformes rays were abundantly landed, especially *G*. *granulatus and G*. *obtusus* followed by *R*. *ancylostoma* and *Rhinobatos ranongensis R*. *ranongensis* was not frequent. These species were frequently targeted using non-baited longlines due to high fin price and meat consumption [24]. Although G. *granulatus*, *R*. *ancylostoma*, *G*. *typus* and *R*. *djiddensis (*probably *R*. *lavies* or *R*. *australie*, as *R*. *djiddensis* doesn’t occur in this region) were commonly previously reported [34, 35, 77–80, 108], the current study reported no *Rhynchobatus* spp., presumably due to extreme population decline. A potential population depletion is corroborated by fishers, who commonly referred to as a white-spotted guitarfish, which is not found anymore; however, a more comprehensive investigation is required to confirm this. In the face of the rapid global population decline of up to 99% of giant guitarfish and wedgefish [24], Bangladeshi waters serve as globally significant habitats [109]. Throughout the study period, a total of 33 largetooth sawfish was also recorded, indicating the landing is higher than documented previously by Haque et al., 2020, and needs immediate conservation action [110]. It is likely that the occurrence of highly vulnerable rays, such as the largetooth sawfish, is unreported in most recent studies as incidental by-catch does not land in the formal landing sites [110].

### 4.3. Relative aggregate landings

Our study found Bangladesh’s substantial catch on elasmobranchs in the Bay of Bengal region. However, the previously accounted amounts were vastly conservative, leading to sizeable unreported catch. The findings reveal aggregate landings of elasmobranchs in Bangladeshi landing sites reported in the national statistics are a conservative estimate corroborated by other studies [70]. Bangladesh could not report a significant share of its elasmobranch catch due to the lack of monitoring mechanism in place in fishing vessels, informal landings at sea beaches and formal landing sites. For instance, according to the Bangladeshi national statistics, the elasmobranch landing was 4496 t on an average annually from 2016–17 [69]. However, it was reported to be much higher (between 8000 and 19600 t) through reconstruction studies and field observations [Haque unpubl. data, 41, 61].

Moreover, FAO ranked Bangladesh as the 19th country by volume of fin trade export with an average export of 95 tonnes of shark fins from 2000–11 [25]. Both of these are conservative estimates due to the scale of IUU fishing, the actual catch being 3–4 times higher than the reported landings [25]. This is reflected in flawed international datasets as well. For instance, one reconstruction study has reported that marine landing is 157% higher than the numbers reported by FAO [70]. This reconstruction study based on data from 1993 to 2007 revealed that between 7000 t and ~ 11000 t of elasmobranchs were caught from Bangladeshi waters. Between 2008 and 2016, the numbers fluctuated between 5500 t to 8500 t [41]. It is worth noting that the numbers in several years were higher than Peru, Korea, Yemen and Ecuador, which are amongst the top 20 elasmobranch catchers in the world [1]. The increase in the catch since the 1950s is related to the increasing fishing pressure.

Additionally, fishing efforts increased at least four-fold between 2000 and 2014 for Bangladesh [41]. Bangladeshi total elasmobranch catch contributes at least 4% of total elasmobranch catches in the Bay of Bengal region, much higher than the Maldives and Thailand (from Andaman sea). This percentage of catch is also very close to Sri Lanka, Thailand (Gulf areas), Gujarat, the third-highest harvester of India [32, 33] and Peninsular west of Malaysia [41], again EEZ of several of which are much higher than Bangladesh within the Bay of Bengal region. This indicates either higher efforts in a comparatively smaller region like Bangladesh or greater population aggregation in the very productive Ganges basin region of the Bay of Bengal region.

### 4.4. Threats from unmanaged artisanal fisheries

The results of this study show that juvenile sharks and rays are caught in abundance. A high proportion of immature individual catch in fisheries is a clear indicator of unsustainable fisheries [101, 111]. Although in many fisheries targeting smaller immature individuals is a tactic for sustainability [112] due to greater catch rates, higher meat quality or lower mercury content [101], this is not the case in Bangladesh. Fishers in Bangladesh unselectively catch both larger and smaller elasmobranchs. In the absence of local length at maturity data (Lm), the life history traits of elasmobranchs from other regions indicate that an incredible number of Bangladeshi fished species are immature and caught before reproducing. This was mainly for *S*. *lewini*, *C sorrah*, *C*. *limbatus*, *C*. *amboinensis*, *G*. *cuiver*, *G*. *granulatus;* even the most frequently caught *S*. *laticaudus*. The case was similar for rays.

Although a large proportion of immature catch can be part of a well-managed fishery [113], this is not the case for Bangladesh, which has no current catch limits or protection of any size class. As a result, both juveniles and reproducing adults, including gravid females, are unselectively caught. The depletion of the mature size classes in the catch indicates unsustainable fisheries as this suggests that size composition has shifted toward smaller individuals over time due to excessive fishing pressure [101]. This is corroborated by fishers interviews whereby fishers identified depletion of larger sharks (both number and size) and rays over the last decade, especially hammerhead sharks (*Sphyrna* spp.), bigger charcharinids, whale sharks, guitarfishes (*Rhynchobatus* spp., *Glaucostegus* spp., *Rhinobatos* spp.) attributed to overfishing and increased bottom trawling [Haque unpubl. data, 109]. The dominance of immature individuals in the catch of elasmobranchs is due to increased fishing pressure is a common phenomenon in many parts of the world (e.g. Indonesia, Costa Rica) [101, 114] as a result of unmanaged fisheries.

A substantial number of juveniles were recorded for these species including gravid females especially in winter, pre-monsoon season. This information can be used for informed decision-making regarding seasonal closure and size-class ban for sustainable management. Almost all types of fishing gear catch sharks and rays. The catchability of smaller sharks is also high in the gear used in this region as an array of mesh sizes are reported exploiting different water columns [78, 79]. Whereas monofilament gill nets were most destructive for smaller sized sharks, the larger ones were caught in an array of large mesh sized nets. Additionally, targeted ray long line hooks, bottom set bamboo nets, and set-bag nets are most destructive for all demersal rays, including Rhinopristiformes rays. Given the increasing mesh size has a positive relationship with the increasing length of shark catch found in this study and using the gear specific catch pattern, size and gear-dependent management regimes may be appropriate.

Furthermore, the landing of elasmobranchs in Bangladesh has a decreasing trend which poses greater concerns as this could be due to population decline in the Bay of Bengal region. For instance, several countries bordering the Bay of Bengal have reported a steep decline in elasmobranch catch and landing, likely due to the continued increase in fishing efforts. For example, from 15000 t in the 1990s to 40000 t in 2014, the increase in elasmobranch catch in Myanmar is concurrent with an increase in the fishing effort [41]. A ~50% decline is reported in the elasmobranch catch rate between 1978–80 and 2013 [115], with an increase in catch of smaller short-lives species compared to larger long-lived ones, indicating overexploitation of the elasmobranch populations [116, 117]. Similar patterns are evident in Bangladesh, where fishing efforts has increased more than 1300 times in the last 60 years [90] and abundant catch of short-lived species. The decline is confirmed by steep depletion in biomass of elasmobranchs, as well as the average size, number and diversity of the animals landed (Haque in prep.) which has been reported by fishers.

The decline of elasmobranch in such a biodiverse area as the Bay of Bengal is problematic from a global perspective as Bay of Bengal inhabits globally threatened species, including endemic species. Similar or more extreme declines in catch have been recorded globally; for example, an 89% drop in elasmobranch landings was recorded in Thailand between 2003–2018 [118] and 67% decline in China over the 65 years [119], having similar fishing pressure as Bangladesh. Consistently, both the east and west coast of the Malaysian Peninsular reported a decline of 30% and 54% in 2014.

As an economically impoverished country in the Bay of Bengal region, Bangladesh has not had capacity to support species conservation adequately. In general, there is evidence that poorer countries with large populations and high elasmobranch product export, report particularly steep declines in elasmobranchs [120]. For instance, Vietnam reported a 97% decline in landings over 29 years from 1986–2014 and Cambodia reporting a 91% reduction over 12 years from 2003–2014 [41]. These declines were attributed directly to fishing pressure [120]. In contrast, U.S. shark fisheries are considered as some of the most sustainable in the world with observed population growth in some commercially important species (the spiny dogfish fishery, on the U.S. North Atlantic coast, population of which rebuilt since 2010) [121, 122]. This is because of the availability of resources to implement robust science-based management.

Overall, the decreased fishing effort can cause a decline in the catch; thus, it is difficult to infer the population trend from catch or landing data. However, with an increase in fishing effort, these declines indicate reductions in elasmobranch populations, which should be cause for concern [41].

The fishing effort in Bangladesh increased from 420 to 582670 kW overall between 1950 and 2014 [70]. The effort increased four-fold between 2000 and 2014 [41, 90]. Studies have shown, the average landings of elasmobranch have declined from the 1990s [41, 70] with a reduction in the composition, size and number of elasmobranchs in Bangladeshi waters. Like other Bay of Bengal countries mentioned here, these indicate population decline at sea over a long time.

### 4.5. Conservation challenges

A high proportion of the species recorded in this study are threatened with extinction, according to the IUCN Red List. However, while IUCN assessments include species found in the Bay of Bengal, they lack regional risks and threats information and require updating in a regional context. This study can help address this, providing regional data to underpin the assessments, reliable data is a pre-requisite for management [101, 114, 123]. Furthermore, although Bangladesh is a signatory for both the CITES and the CMS, implementation and enforcement are lacking. Bangladesh national law only protects a total of 29 elasmobranch species under the Wildlife (Conservation and Security) Act, 2012, omitting eleven CITES species. There is a clear need to amend and expand the single act protecting vulnerable species in Bangladesh and at the same time increase the enforcement of relevant laws.

Several species of elasmobranchs have depleted in the Bay of Bengal region (e.g. sawfish, wedgefish- *Rhynchobatus* spp., winged hammerhead shark, great hammerhead shark, and possibly many others have depleted unnoticed) to such critical levels that rebuilding them requires urgent action and may take a long time [124]. However, species-specific and trade-based legislation alone will not be enough to protect elasmobranchs in this region. Specific management measures pertaining to elasmobranch fisheries are also needed. Given that fishing is of high importance to the livelihoods of millions of fishers in Bangladesh and has a significant role in the country’s economy [50], fisheries management needs to ensure sustainability for elasmobranch stocks, as well as maintaining livelihoods. It is particularly significant as the global demand for protein [125] has increased and collapse of global fisheries in many cases are evident [126]. Species-specific sustainable fisheries approaches, with an acute understanding of needs and actions based on robust evidence-based strategies [127], can be viable.

Strategies include size or catch limits or live release of bycatch. Size or catch limit may be ineffective if there is a lack of understanding about stocks [128]; the success of live release is also dependent on post-release mortality and its effects on the species [129], indicating more research is needed. While doing so, Bangladesh’s main challenge will be to ensure pre-cautionary and proactive approaches for policies, implementation, and enforcement of laws. This is because the late global response and reactive approach towards saving depleted species have led to complicated conservation scenarios [130]. In many cases, they have merely documented the depletion without acting. To maintain and conserve elasmobranch populations, this needs to change immediately, with proactive, evidence-based and rapid measures.

### 4.6. Further research

Identifying species at landing and trading sites is a challenging task. The absence of national species lists and guides sometimes with invalid/misapplied names [36] and poorly curated reference collections also make identification challenging, often with reliance on regional and global identification guides. However, through this study, the capacity for field identification of morphologically similar sharks and rays has been improved with reference photographs and genetic sampling (Haque, unpubl. data), such as for *Maculabatis* spp. *Mobula* spp., and *Neotrygon* spp. amongst many. The study found several challenges of species identification at field sites that needs to addressed urgently by further research. Taxonomic problems need resolving for many elasmobranch species, with a large number of descriptions by earlier ichthyologists recently synonymised [12, 86, 87] or not yet identified to species level (e.g. *Iago* spp. and *Narcine* sp. [21, 131]. The identification of morphologically different or geographic sub-populations with endemic or cryptic species needs further work. With the possibilities of geographically isolated population variants and species new to science, different variants are currently being studied for better taxonomic understanding.

The findings of this study will greatly improve the information required to underpin the conservation and management of elasmobranchs in the region. To conclusively resolve elasmobranch taxonomy, more extensive geographic sampling may be required [87, 132–136] and in conjunction with genetic and morphological sampling (e.g. for Carcharhiniformes, *Neotrygon* spp., many Dasyatids, *Iago* spp.*)*. Such an approach could lead to the discovery of greater diversity in the Indian Ocean, particularly Bangladesh, which is amongst the most understudied regions [23, 104]. Genetic studies may also be vital in better understanding the trade in elasmobranch products [11, 21, 26, 71, 83, 84, 137–140]. Financial resource limitation can often prevent such studies from occurring [140] meaning that molecular methods are often not used at scale [27]; hence more collaborative approaches are required.

### 4.7. Recommendations

**Fig 11:**
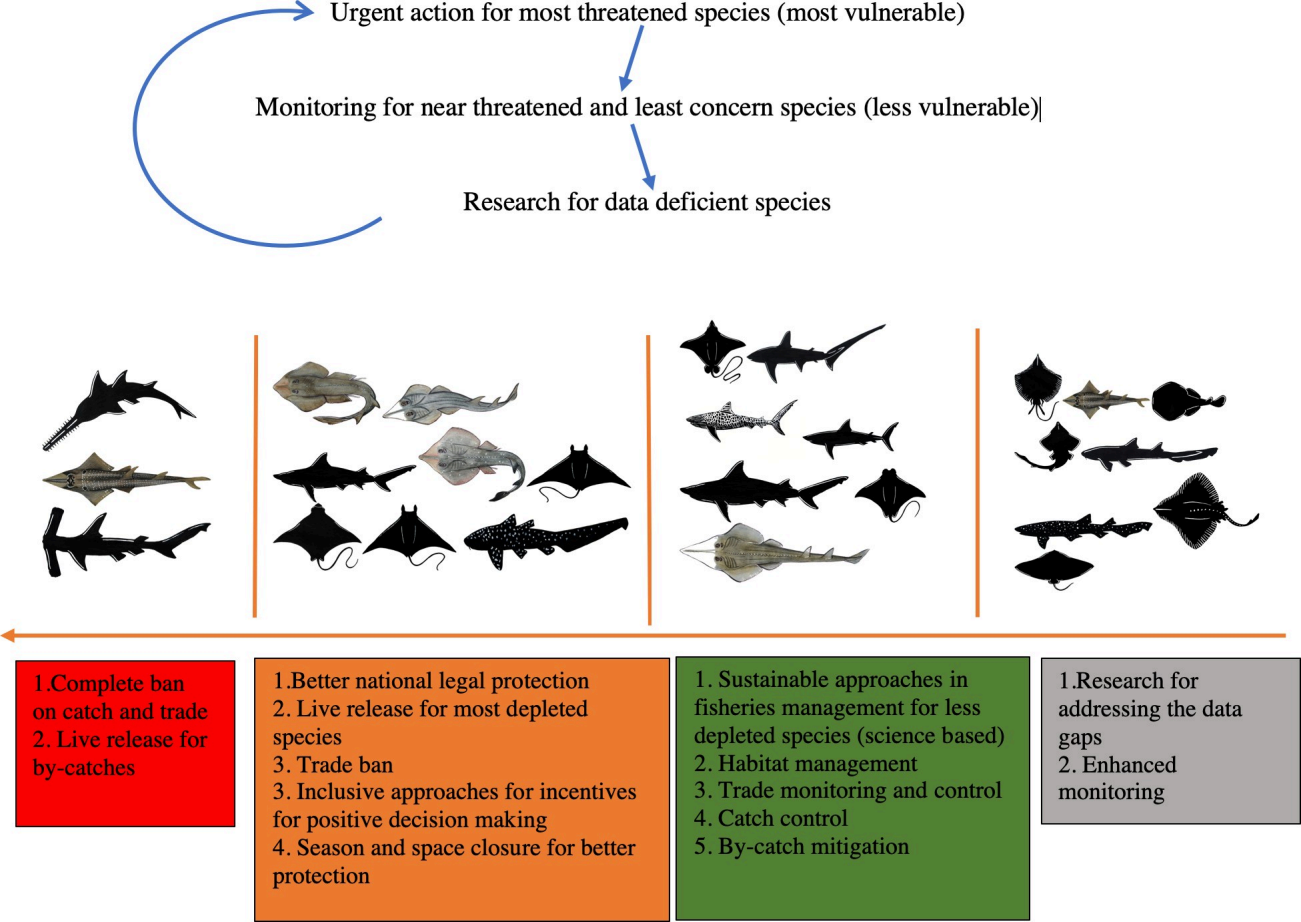
Specific conservation and research recommendations for different group of animals in accordance with their degree of threat.

Based on the results of this study, Table 3 presents a series of recommendations for enhancing the conservation status of elasmobranchs in Bangladesh. These recommendations are rigorously prepared and was in accordance with the International Plan of Action for the conservation and management of sharks [23, 141] and classified into immediate actions for the most threatened taxa; a sustainable conservation approach for less vulnerable taxa; and further research combined with a precautionary approach for data-deficient taxa (Fig 11).

**Table 3:** Priority recommendations for elasmobranch research and conservation actions.

Stakeholders	Recommendations
Government/ state authoritative bodies	1. Amend national legislation to protect the most vulnerable species by banning catch and trade, and instating catch limits, size limits, and areal and/or seasonal closures for those capable of some level of fisheries;
	2. Ensure complete protection for species assessed as CR and EN by the IUCN Red List;
	3. Enhance implementation and compliance towards the mandates of international agreements, including CITES and CMS, and the national law;
	4. Improve coordination with affiliated government departments for managing coastal and marine waters;
	5. Implement an improved accounting system for both landing sites and fishing vessels (e.g. logbooks, vessel trackers, observers, trained accountants in the landing sites) for transparent catch estimations and improved monitoring;
	6. Commit to sustained political will and regional cooperation to conserve elasmobranchs in this region;
	7. Support a regional Red List assessment for the Bay of Bengal.
Practitioners	1. Develop a National Action Plan for elasmobranchs whereby all stakeholders, including the fishers, can participate to devise acceptable and implementable actions towards conservation of elasmobranchs in this region and work with the government to implement that;
	2. Work in tandem with researchers and government bodies for better implementation of conservation measures;
	3. Develop nationwide public educational programs especially for fishers and traders.
Researchers	1. Establish techniques for by-catch reduction, including through gear and mesh size selectivity, and encourage proper handling and live release where necessary;
	2. Combine taxon specific biological (e.g. breeding biology and season) and ecological studies (e.g. trophic level interactions) for better understanding of populations and the intrinsic and extrinsic threats;
	3. Enhance taxonomic research and local research capacity through universities and research institutes;
	4. Conduct habitat level studies;
	5. Understand locally contextualised vulnerabilities to help define priorities for elasmobranch conservation.

These recommendations will be most effective if implemented as a long-term plan for the region. Key to success is enhanced coordination among researchers, practitioners and stakeholders, collaboration with neighbouring nations for better-coordinated policies and actions based on research.

## 5. Conclusion

This study has provided up to date knowledge on the species richness and distribution of elasmobranchs in the Bangladeshi Bay of Bengal and confirmed the identification of various morphologically similar and previously misidentified and cryptic species. The findings highlight that elasmobranch protection in Bangladesh is not adequate and in the absence of regional IUCN Red List assessments and understanding of the extinction risk, this work contributes to the knowledge base for prioritising actions for vulnerable species. The results highlight the urgent need to improve conservation and fisheries management within the Bay of Bengal, as well as globally. Urgent interventions are needed before unmonitored catch and trade further deplete elasmobranch stocks to the point where it becomes irreversible. While more species-specific studies are needed, immediate inclusive conservation measures are urgently recommended. These results can be used for identifying priority groups for immediate conservation action, and for amending the national act to provide enhanced protection in line with international agreements such as CITES and CMS. The fate of elasmobranchs in the Bay of Bengal depends on all stakeholders’ individual and collective efforts and, ultimately, the political will of all surrounding nations. Regional fisheries management organisations can act towards further and better coordination in managing pelagic and migratory species. Reducing fishing pressures and habitat degradation by bottom trawling in coastal areas is crucial in this regard and should be given top priority. These can be achieved by enhanced law enforcement and local communities’ capacity building towards sustainable fishing ensuring better livelihood options. Finally, this study puts the Bay of Bengal, Bangladesh, on the global seascape map as a priority area for the conservation of vulnerable elasmobranch species.

## Supporting information

S1 Fig Habitat uses. Habitat and ecological niche of each species within each family of elasmobranchs reported in the checklist.
(TIF)

S2 Fig Species in each family reported in the checklist. Blue shows the species occurrence from the region was confirmed and evaluated following recent publications and globally accepted range studies and red stands for species needed further confirmation.
(TIF)

S3 Fig Landings of elasmobranchs from the Bay of Bengal, Bangladesh from 1950–2016 from the data obtained from Sea Around Us. (TIF)

S1 Table Annotated checklist of elasmobranchs in Bangladesh (Until June 2020). (DOCX)

S2 Table Relative landings of aggregate elasmobranchs in the Bay of Bengal countries from 1950–2016 with and mean, standard deviation and percentage of total catch contribution from the Bay of Bengal areas. (DOCX)

S3 Table Observation on the abundance of different elasmobranch species in landing, plausible reasons and conservation implications. (DOCX)
