# Supplementary figures and images for: Evaluating artisanal fishing of globally threatened sharks and rays in the Bay of Bengal, Bangladesh
Source: PLoS One. 2021 Sep 9;16(9):e0256146. doi: 10.1371/journal.pone.0256146 (PMC8428726; doi:10.1371/journal.pone.0256146)

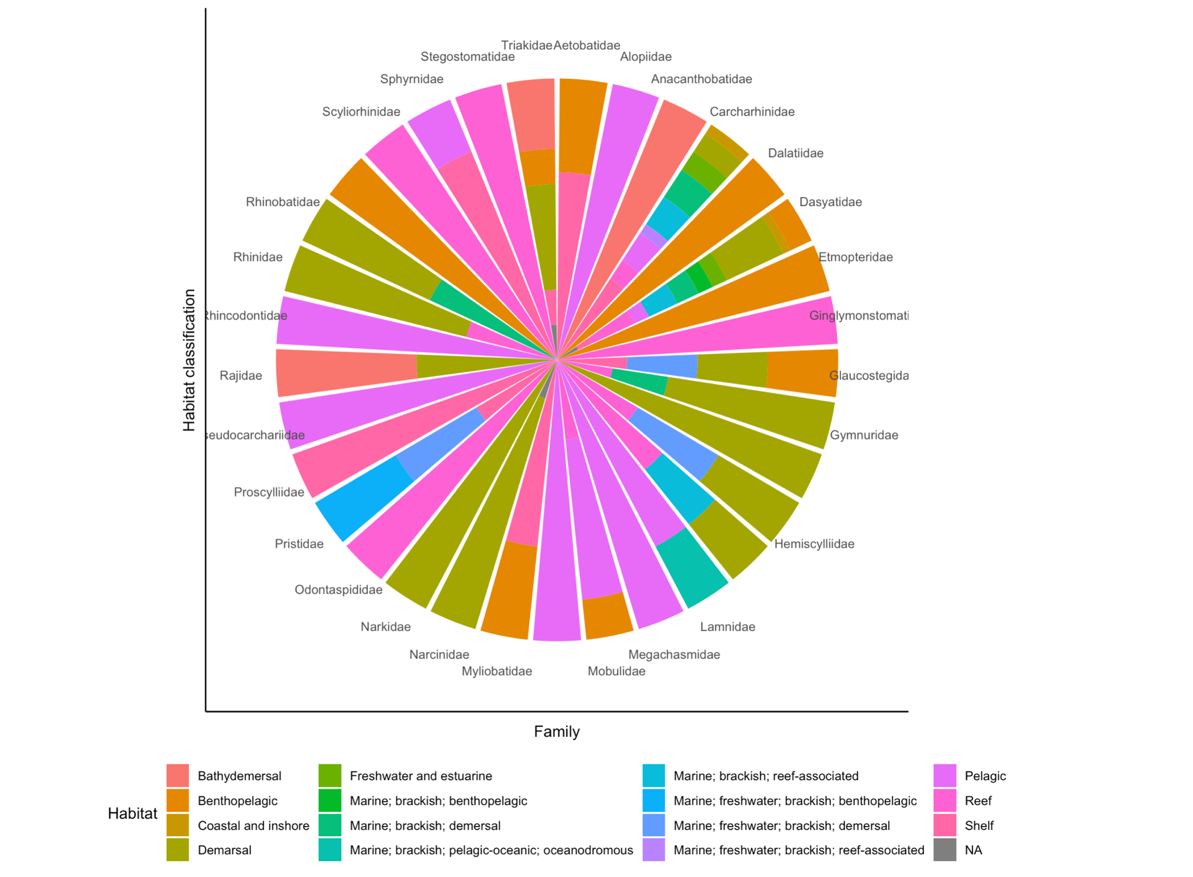

Supplement: S1 Fig — Habitat and ecological niche of each species within each family of elasmobranchs reported in the checklist. (TIF) [file pone.0256146.s001.tif]

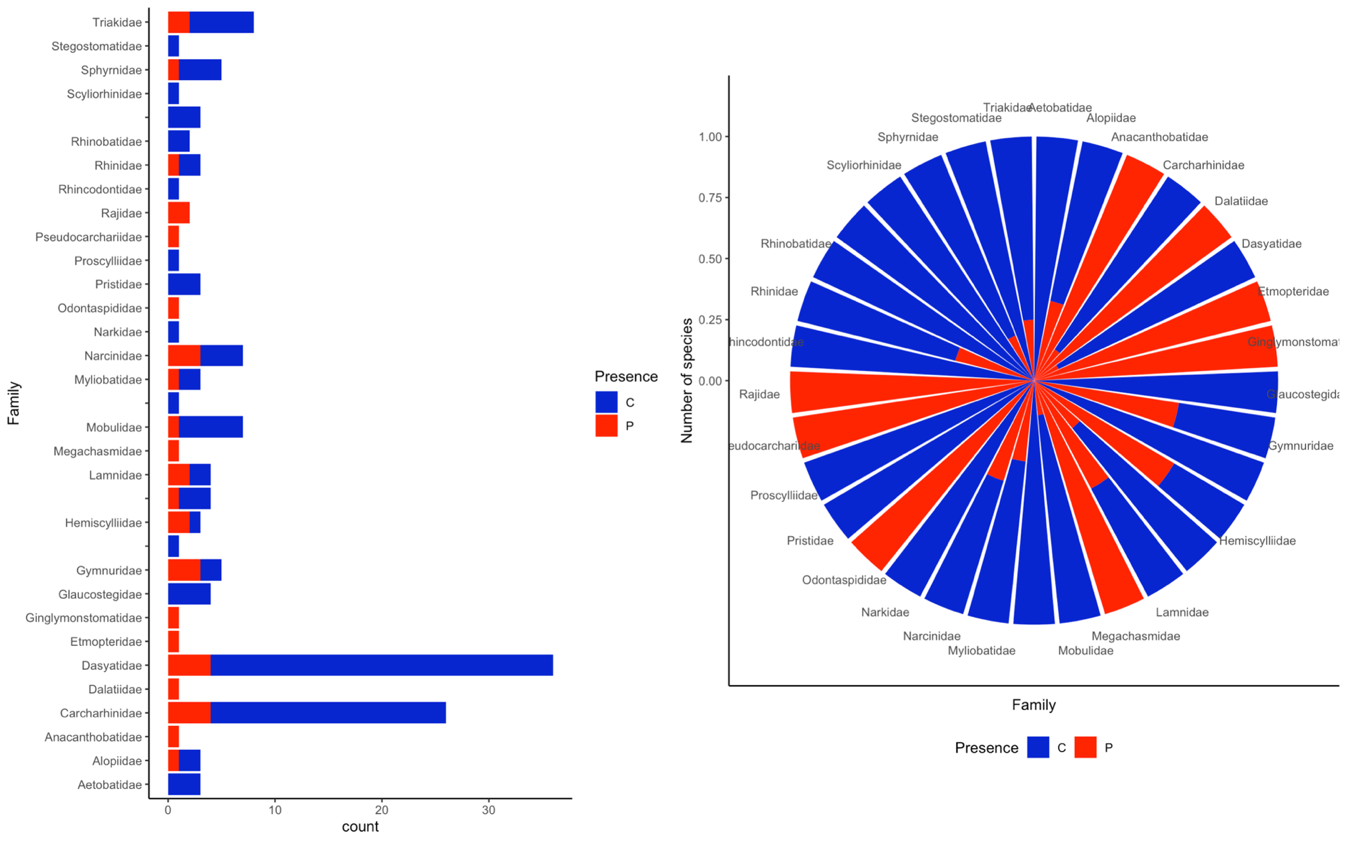

Supplement: S2 Fig — Blue shows the species occurrence from the region was confirmed and evaluated following recent publications and globally accepted range studies and red stands for species needed further confirmation. (TIF) [file pone.0256146.s002.tif]

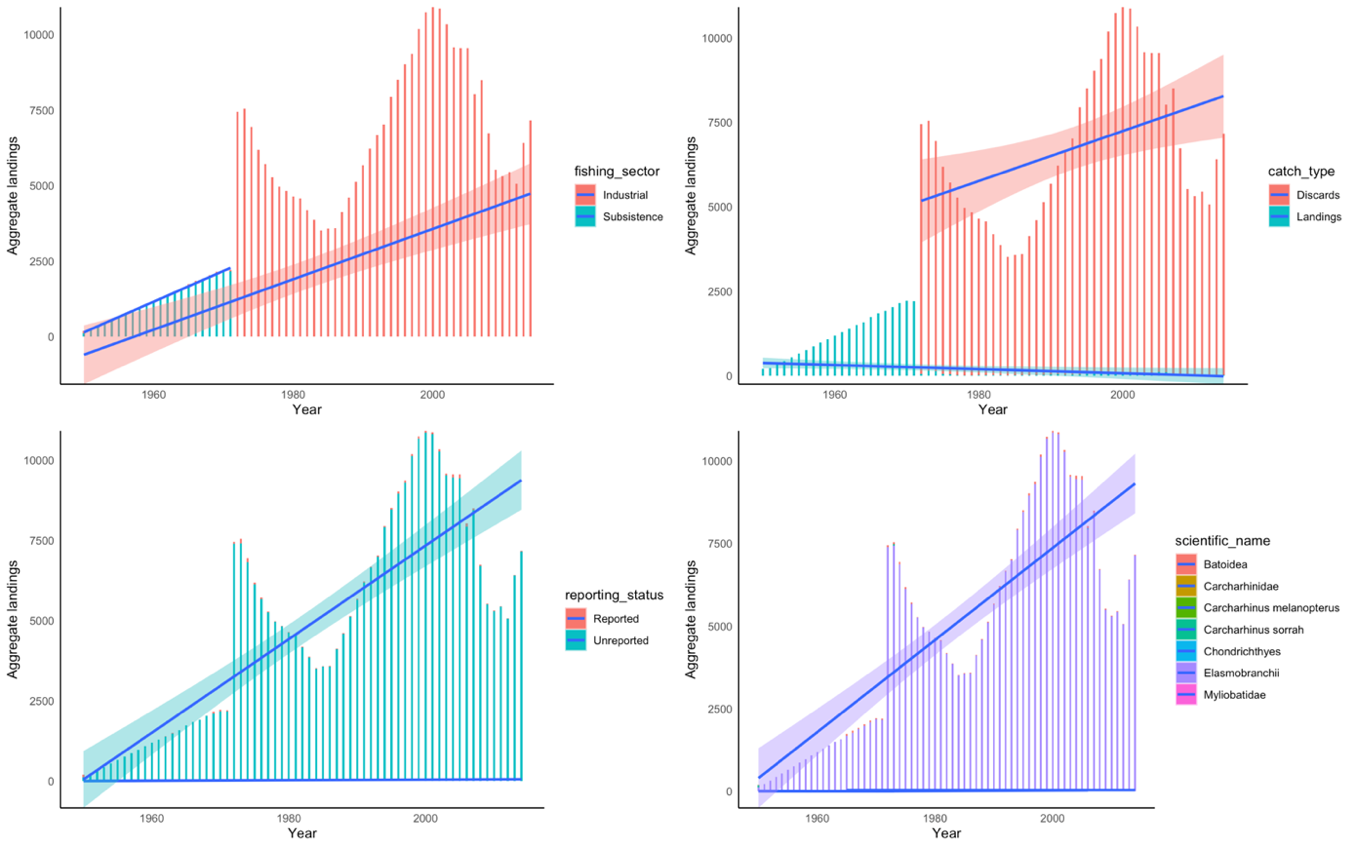

Supplement: S3 Fig — (TIF) [file pone.0256146.s003.tif]
