# Supplementary material for: Evaluating artisanal fishing of globally threatened sharks and rays in the Bay of Bengal, Bangladesh
Source: PLoS One. 2021 Sep 9;16(9):e0256146. doi: 10.1371/journal.pone.0256146 (PMC8428726; doi:10.1371/journal.pone.0256146)
Supplement: S1 Table — (DOCX) [file pone.0256146.s004.docx]

**S1 Table.** Annotated checklist of elasmobranchs in Bangladesh (Till June 2020).

| Sharks | | | | | | | | |
| --- | --- | --- | --- | --- | --- | --- | --- | --- |
| Family | **Reported Name** | **Valid scientific name** | **Common name** | **Comments** | **IUCN** | **CITES (App.)** | **CMS (App.)** | **National Protection** |
|  | *Hypoprion palasorrah* |  |  | Questionable identity: was reported by Hossain et al., 1970 | - | - | - | - |
| Alopiidae | *Alopias pelagicus* | *Alopias pelagicus* | Thresher Shark | Reported from Bay of Bengal, Confirmation needed for Bangladesh | EN | II | II | - |
| Alopiidae | *Alopias superciliosus* | *Alopias superciliosus* | Bigeye thresher | CITES trade database | VU | II | II | - |
| Alopiidae | *Alopias vulpinus* | *Alopias vulpinus* | Common Thresher | CITES trade database | VU | II | II | - |
| Carcharhinidae | *Carcharhinus amblyrhynchoides* | *Carcharhinus amblyrhynchoides* | Graceful Shark | Confirmed presence | NT | - | - | - |
| Carcharhinidae | *Carcharhinus amboinensis* | *Carcharhinus amboinensis* | Pigeye Shark/ Java shark | Confirmed presence | DD | - | - | - |
| Carcharhinidae | *Carcharhinus brevipinna* | *Carcharhinus brevipinna* | Spinner Shark | Confirmed presence | VU | - | - | - |
| Carcharhinidae | *Carcharhinus dussumieri* | *Carcharhinus dussumieri* | Whitecheek shark | Confirmed presence | EN | - | - | I |
| Carcharhinidae | *Carcharhinus menisorrah* | *Carcharhinus falciformis* | Silky shark | Confirmed presence | VU | II | II | I |
| Carcharhinidae | *Carcharhinus hemiodon* | *Carcharhinus hemiodon* | Pondicherry shark | Fishbase and IUCN distribution map shows the range includes Bangladeshi waters. | CR | - | - | - |
| Carcharhinidae | *Carcharhinus leucas* | *Carcharhinus leucas* | Bull Shark | Confirmed presence | NT | - | - | - |
| Carcharhinidae | *Carcharhinus limbatus* | *Carcharhinus limbatus* | Blacktip Shark | Confirmed presence | NT | - | - | I |
| Carcharhinidae | *Carcharhinus longimanus* | *Carcharhinus longimanus* | Oceanic whitetip shark | CITES trade database | CR | II | I | - |
| Carcharhinidae | *Hypoprion macloti* | *Carcharhinus macloti* | Hardnose shark | Confirmed presence | NT | - | - | I |
| Carcharhinidae | *Carcharhinus melanoptera* | *Carcharhinus melanopterus* | Blacktip reef shark | Confirmed presence | VU | - | - | - |
| Carcharhinidae | *Carcharhinus sealei* | *Carcharhinus sealei* | Blackspot shark | Fishbase and IUCN distribution map shows the range includes Bangladeshi waters. | NT | - | - |  |
| Carcharhinidae | *Carcharhinus sorrah* | *Carcharhinus sorrah* | Spot-tail shark | Confirmed presence | NT | - | - | I |
| Carcharhinidae | *Carcharhinus ellioti* | *Carcharius ellioti* / *Hemipristis elongata* | Snaggletooth shark | Confirmed presence | VU | - | - | - |
| Carcharhinidae | *Galeocerdo cuvier* | *Galeocerdo cuvier* | Tiger shark | Confirmed presence | NT | - | - | I |
| Carcharhinidae | *Carcharhinus gangetica* | *Glyphis gangeticus* | Ganges shark | Confirmed presence | CR | - | - | I |
| Carcharhinidae | *Glyphis glyphis* | *Glyphis glyphis* | Speartooth Shark | Need further confirmation | EN | - | - |  |
| Carcharhinidae | *Lamiopsis temminckii* | *Lamiopsis temminckii* | Broadfin Shark | Confirmed presence | EN | - | - | - |
| Carcharhinidae | *Loxodon macrorhinus* | *Loxodon macrorhinus* | Sliteye shark | Confirmed presence | LC | - | - | - |
| Carcharhinidae | *Negaprion acutidens* | *Negaprion acutidens* | Sicklefin lemon shark | Need further confirmation | VU | - | - | - |
| Carcharhinidae | *Prionace glauca* | *Prionace glauca* | Blue shark | Need further confirmation | NT | - | II | - |
| Carcharhinidae | *Scoliodon walbeehmii* | *Rhizoprionodon acutus* | Milk Shark | Confirmed presence | VU | - | - | I |
| Carcharhinidae | *Rhizoprionodon oligolinx* | *Rhizoprionodon oligolinx* | Grey sharp nose shark | Confirmed presence | LC | - | - | - |
| Carcharhinidae | *Physodon mulleri* | *Scoliodon laticaudus* | Spadenose Shark | Synonym of Scoliodon laticaudus | NT | - | - | - |
| Carcharhinidae | *Scoliodon sorrakowah* | *Scoliodon laticaudus* | Spadenose shark | Confirmed presence | NT | - | - | I |
| Carcharhinidae | *Scoliodon macrorhynchos* | *Scoliodon macrorhynchos* | Pacific spadenose shark | Confirmed presence | NT | - | - | - |
| Carcharhinidae | *Triaenodon obesus* | *Triaenodon obesus* | Whitetip reef shark | Confirmed presence | VU | - | - | - |
| [Dalatiidae](https://www.fishbase.se/summary/FamilySummary.php?ID=557) | *Euprotomicrus bispinatus* | *Euprotomicrus bispinatus* | Pygmy shark | Circum-global range according to Fishbase weebsite. | LC | - | - | - |
| Etmopteridae | *Centroscyllium ornatum* | *Centroscyllium ornatum* | Ornate dogfish | Need further confirmation | LC | - | - | - |
| Ginglymonstomatidae | *Nebrius ferrugineus* | *Nebrius ferrugineus* | Tawny nurse shark | Need further confirmation | VU | - | - | - |
| Hemigaleidae | *Hemigaleus balfouri* | *Chaenogaleus macrostoma* | Hooktooth shark | Confirmed presence | VU | - | - | - |
| Hemiscylliidae | *Chiloscyllium burmensis* | *Chiloscyllium burmensis* | Burmese Bamboo Shark | Confirmed presence | VU | - | - | - |
| Hemiscylliidae | *Chiloscyllium* cf*. arabicum* | *Chiloscyllium* cf. *arabicum* | Arabian carpetshark | Need further confirmation | NT | - | - | - |
| Hemiscylliidae | *Chiloscyllium griseum* | *Chiloscyllium griseum* | Grey bambooshark | Confirmed presence | VU | - | - | I |
| Hemiscylliidae | *Chiloscyllium hasseltii* | *Chiloscyllium hasselti* | Hasselt's bambooshark | Confirmed presence | EN | - | - | - |
| Hemiscylliidae | *Chiloscyllium indicum* | *Chiloscyllium indicum* | Slender bambooshark | Confirmed presence | VU | - | - | - |
| Hemiscylliidae | *Chiloscyllium plagiosum* | *Chiloscyllium plagiosum* | Whitespotted bamboo shark | Fishbase and IUCN distribution map shows the range includes Bangladeshi waters. | NT | - | - | - |
| Hemiscylliidae | *Chiloscyllium punctatum* | *Chiloscyllium punctatum* | Grey Carpetshark | Need further confirmation | NT | - | - | I |
| Lamnidae | *Lamna nasus* | *Lamna nasus* | Porbeagle shark | CITES trade database | VU | II | II | - |
| Lamnidae | *Carcharodon carcharias* | *Carcharodon carcharias* | Great white shark | Cosmopolitan distribution according to Fishbase | VU | II | I & II | - |
| Lamnidae | *Isurus oxyrinchus* | *Isurus oxyrinchus* | Shortfin mako | Confirmed presence | EN | II | II | - |
| [Lamnidae](https://www.fishbase.se/summary/FamilySummary.php?ID=9) | *Isurus paucus* | *Isurus paucus* | Longfin mako | Circumglobal range according to Fishbase and IUCN | EN | II | II | - |
| [Megachasmidae](https://www.fishbase.se/summary/FamilySummary.php?ID=519) | *Megachasma pelagios* | *Megachasma pelagios* | Megamouth shark | Circumglobal range according to Fishbase and IUCN | LC | - | - | - |
| [Odontaspididae](https://www.fishbase.se/summary/FamilySummary.php?ID=8) | *Carcharias taurus* | *Carcharias taurus* | Sand tiger shark | Circumtropical according to Fishbase | VU | - | - | - |
| Proscylliidae | *Eridacnis radcliffei* | *Eridacnis radcliffei* | Pigmy ribbontail catshark | Confirmed presence | LC | - | - | - |
| [Pseudocarchariidae](https://www.fishbase.se/summary/FamilySummary.php?ID=556) | *Pseudocarcharias kamoharai* | *Pseudocarcharias kamoharai* | Crocodile shark | Tropical and subtropical waters of all oceans according to Fishbase | LC | - | - | - |
| Rhincodontidae | *Rhincodon typus* | *Rhincodon typus* | Whale Shark | Confirmed presence | EN | II | I & II | I |
| [Scyliorhinidae](https://www.fishbase.se/summary/FamilySummary.php?ID=10) | *Atelomycterus marmoratus* | *Atelomycterus marmoratus* | Coral catshark | Confirmed presence | NT | - | - | - |
| Sphyrnidae | *Sphyrna blochii* | *Eusphyra blochii* | Winghead shark | Confirmed presence | EN | - | - | I |
| Sphyrnidae | *Sphyrna lewini* | *Sphyrna lewini* | Scalloped Hammerhead | Confirmed presence | CR | II | II | I |
| Sphyrnidae | *Sphyrna mokarran* | *Sphyrna mokarran* | Great hammerhead shark | Confirmed presence | CR | II | II | - |
| Sphyrnidae | *Sphyrna tudes* | *Sphyrna tudes* | Smalleye hammerhead | Questionable record. Distributed through mainly Western Atlantic and eastern Pacific; Mediterranean Sea | CR | - | - | - |
| Sphyrnidae | *Sphyrna zygaena* | *Sphyrna zygaena* | Smooth hammerhead | Confirmed presence | VU | II | II | I |
| Stegostomatidae | *Stegostoma fasciatum* | *Stegostoma fasciatum/ Stegostoma tigrinum* | Zebra shark | Confirmed presence | EN | II | - | I |
| Triakidae | *Galeorhinus galeus* | *Galeorhinus galeus* | Tope shark | Questionable record. A temperate species. | CR | - | II | - |
| Triakidae | *Iago garricki* | *Iago garricki* | Longnose Houndshark | Confirmed presence | LC | - | - | - |
| Triakidae | *Iago* cf. *omanensis* | *Iago omanensis* | Bigeye houndshark | Confirmed presence | LC | - | - | - |
| Triakidae | *Iago sp.* | *Iago* cf. *omanensis* | Bigeye houndshark | Need further confirmation | LC | - | - | - |
| Triakidae | *Mustelus griseus* | *Mustelus griseus* | Spotless Smoothhound | Confirmed presence | EN | - | - | - |
| Triakidae | *Mustelus kanekonis* | *Mustelus kanekonis* | Spotless Smoothhound | Confirmed presence | NE | - | - | I |
| Triakidae | *Mytmillo manazo* | *Mustelus manazo* | Starspotted smooth-hound | Confirmed presence | EN | - | - | - |
| Triakidae | *Mustelus mosis* | *Mustelus mosis* | Arabian smooth-hound | Confirmed presence | NT | - | - | - |
| Rays | | | | | | | | |
| [Anacanthobatidae](https://www.fishbase.se/summary/FamilySummary.php?ID=524) | *Sinobatis andamanensis* | *Sinobatis andamanensis* | Andaman Legskate | Bay of Bengal is a range according to the maps of Last et al., 2016. Possible presence | LC | - | - |  |
| Aetobatidae | *Aetobatus flagellum* | *Aetobatus flagellum* | Longheaded Eagle Ray | Confirmed presence | EN | - | - | - |
| Aetobatidae | *Aetobatus ocellatus* | *Aetobatus ocellatus* | Ocellated eagle ray | Confirmed presence | VU | - | - | - |
| Aetobatidae | *Aetobatus narinari* | *Aetobatus narinari* | Spotted Eagle Ray | Confirmed presence | NT | - | - | II |
| Dasyatidae | *Brevitrygon heterura* | *Brevitrygon heterura* | Dwarf Whipray | Confirmed presence | NE | - | - | - |
| Dasyatidae | *Dasyatis (Amphotistius) imbricata* | *Brevitrygon imbricata* | Scaly Whipray | Confirmed presence | DD | - | - | - |
| Dasyatidae | *Dasyatis (Himantura) walga* | *Brevitrygon walga* | Dwarf Whipray | Confirmed presence | NT | - | - | - |
| Dasyatidae | *Dasyatis (Amphotistius) zugei* | *Dasyatis zugei* | Pale-Edged Stingray | Confirmed presence | NT | - | - | - |
| Dasyatidae | *Dasyatis sinensis* | *Hemitrygon sinensis* | Chinese Sting Ray | Confirmed presence | EN | - | - | - |
| Dasyatidae | *Dasyatis bennettii* | *Hemitrygon bennettii* | Bennett's Cowtail, Bennett's Stingray | Confirmed presence | VU | - | - | II |
| Dasyatidae | *Dasyatis (Himantura) bleekeri* | *Himantura bleekeri/ Himantura uarnacoides* | Bleeker's Whipray | Confirmed presence | EN | - | - | - |
| Dasyatidae | *Himantura fluviatilis* | *Himantura fluviatilis / Pastinachus sephen* | Ganges Stingray/ Cowtail Ray | Synonym by IUCN, separate spp. by Fishbase, hence questionable identtity. | NE/ NT | - | - | - |
| Dasyatidae | *Himantura leoparda* | *Himantura leoparda* | Leopard Whipray | Confirmed presence | VU | - | - | - |
| Dasyatidae | *Dasyatis (Amphotistius) marginatus* | *Himantura marginata* | Blackedge Whipray | Not recognised by Last et al., 2016 | NE | - | - | - |
| Dasyatidae | *Himantura tutul* | *Himantura tutul* | Fine-Spotted Leopard Whipray | A separate species by Bosra et al., 2013 and Farnendo et al., 2019. However, synonym of *Himantura uarnak* according to Last et al., 2016. Not a valid species | NE | - | - | - |
| Dasyatidae | *Dasyatis (Himantura) uarnak* | *Himantura uarnak* | Honeycomb Stingray | Confirmed presence | VU | - | - | - |
| Dasyatidae | *Himantura undulata* | *Himantura undulata* | Leopard Whipray | Confirmed presence | EN | - | - | - |
| Dasyatidae | *Maculabatis arabica* | *Maculabatis arabica* | Pakistan/ Arabic Whipray | Confirmed presence | CR | - | - | - |
| Dasyatidae | *Maculabatis bineeshi* | *Maculabatis bineeshi* | Short-Tail Whipray | Confirmed presence | NE | - | - | - |
| Dasyatidae | *Maculabatis macrura* | *Maculabatis macrura* | Sharpnose Whisray | Need further confirmation | EN | - | - | - |
| Dasyatidae | *Himantura pastinacoides* | *Maculabatis pastinacoides* | Round Whip Ray | Confirmed presence | EN | - | - | - |
| Dasyatidae | *Himantura gerrardi* | *Maculabatis gerrardi* | Sharpnose Stingray | Confirmed presence | EN | - | - | - |
| Dasyatidae | *Megatrygon microps* | *Megatrygon microps* | Smalleye Stingray | Confirmed presence | DD | - | - | - |
| Dasyatidae | *Neotrygon* Bay of Bengal varient | *Neotrygon* Bay of Bengal varient |  | Need further studies for taxonomic resolution among morphologically similar species | - | - | - | - |
| Dasyatidae | *Neotrygon caeruleopunctata* | *Neotrygon caeruleopunctata* | Blue Spotted Maskray | Confirmed presence | NE | - | - | - |
| Dasyatidae | *Neotrygon* cf. *caeruleopunctata* | *Neotrygon* cf. *caeruleopunctata* | Bluespotted Maskray | Confirmed presence | NE | - | - | - |
| Dasyatidae | *Neotrygon indica* | *Neotrygon indica* | Blue Spotted Maskray | Confirmed presence | NE | - | - | - |
| Dasyatidae | *Dasyatis (Amphotistius) kuhlii* | *Neotrygon kuhlii* | Blue-Spotted Stingray | Confirmed presence | DD | - | - | II |
| Dasyatidae | *Neotrygon orientalis* | *Neotrygon orientalis* | Oriental bluespotted maskray | Need further confirmation | NE | - | - | - |
| Dasyatidae | *Pastinachus ater* | *Pastinachus ater* | Broad Cowtail Ray | Confirmed presence | LC | - | - | - |
| Dasyatidae | *Pastinachus* cf*. gracilicaudus* | *Pastinachus* cf*. gracilicaudus* | Cowtail Ray | Confirmed presence | - | - | - | - |
| Dasyatidae | *Pastinachus gracillicaudus* | *Pastinachus gracillicaudus* | Narrow Cowtail Ray | Confirmed presence | NE | - | - | - |
| Dasyatidae | *Dasyatis (Pastinachus) sephen* | *Pastinachus sephen* | Cowtail Stingray | Distribution through Northern Indian Ocean; Red Sea to Pakistan | NT | - | - | - |
| Dasyatidae | *Pastinachus solocirostris* | *Pastinachus solocirostris* | Roughnose Stingray | Confirmed presence | EN | - | - | - |
| Dasyatidae | *Himantura fai* | *Pateobatis fai* | Pink Whio Ray | Confirmed presence | VU | - | - | - |
| Dasyatidae | *Himantura jenkinsii* | *Pateobatis jenkinsii* | Jenkins Whipray | Confirmed presence | VU | - | - | - |
| Dasyatidae | *Himantura uarnacoides* | *Pateobatis uarnacoides* | Whitenose Whipray | Confirmed presence | EN | - | - | - |
| Dasyatidae | *Pteroplatytrygon violacea* | *Pteroplatytrygon violacea* | Violate Stingray | Confirmed presence | LC | - | - | - |
| Dasyatidae | *Taeniura lymma* | *Taeniura lymma* | Ribbontail Stingray/Bluespotted Fantail Ray | Confirmed presence | NT | - | - | - |
| Dasyatidae | *Taeniura meyeni* | *Taeniurops meyeni* | Round Ribbontail Ray | Confirmed presence | VU | - | - | - |
| Dasyatidae | *Telatrygon* cf. *crozieri* | *Telatrygon* cf. *crozieri* | Sharpnose Stingray | Need further confirmation | NE | - | - | - |
| Dasyatidae | *Dasyatis zugei* | *Telatrygon zugei* | Pale-Edged Stingray | Confirmed presence | NT | - | - | - |
| Dasyatidae | *Urogymnus africana* | *Urogymnus asperrimus* | Porcupine Ray | Confirmed presence | VU | - | - | - |
| Dasyatidae | *Urogymnus granulatus* | *Urogymnus granulatus* | Mangrove Whipray | Confirmed presence | VU | - | - | - |
| Dasyatidae | *Himantura lobistoma* | *Urogymnus lobistoma* | Tube Mouth Whio Ray | Confirmed presence | EN | - | - | - |
| Dasyatidae | *Urogymnus polylepis* | *Urogymnus polylepis* | Giant Freshwater Stingray | Confirmed presence | EN | - | - | - |
| Gymnuridae | *Gymnura japonica* | *Gymnura japonica* | Japanese Butterfly Ray | Confirmed presence | DD | - | - | - |
| Gymnuridae | *Gymnura micrura* | *Gymnura micrura* | Smooth Butterfly Ray | Questionable record. Distribution mainly Western Atlantic (northern USA to Brazil) and possibly Eastern Atlantic (Senegal to Angola) | DD | - | - | - |
| Gymnuridae | *Gymnura poecilura* | *Gymnura poecilura* | Long-Tailed Butterfly Ray | Confirmed presence | NT | - | - | II |
| [Gymnuridae](https://www.fishbase.se/summary/FamilySummary.php?ID=501) | *Gymnura tentaculata* | *Gymnura tentaculata* | Tentacled Butterfly-Ray | Indo–West Pacific; Red Sea to Bay of Bengal | DD | - | - | - |
| Gymnuridae | *Gymnura zonura* | *Gymnura zonura* | Zonetail Butterfly Ray | Indo–West Pacific; Bay of Bengal (India) to Philippines and Taiwan | VU |  |  | - |
| Mobulidae | *Mobula alfredi* | *Mobula alfredi* | Reef Manta Ray/ Alfred Manta | Need further confirmation | VU | II | I & II | - |
| Mobulidae | *Mobula birostris* | *Mobula birostris* | Giant Manta Ray | Confirmed presence | EN | II | I & II | - |
| Mobulidae | *Mobula diabolus* | *Mobula eregoodootenkee* | Longhorned Mobula | Confirmed presence | EN | II | I & II | - |
| Mobulidae | *Mobula hypostoma* | *Mobula hypostoma* | Lesser Devil Ray | Questionable report as range is: Western and Eastern Atlantic; North Carolina (USA) to northern Argentina, and Mauritania to Angola. | EN | II | I & II | - |
| Mobulidae | *Mobula japanica* | *Mobula mobular* | Spinetail Mobula | Not a separate sp. Conspicific with M. mobular | EN | II | I & II | II |
| Mobulidae | *Mobula kuhlii* | *Mobula kuhlii* | Shortfin Devil Ray | Confirmed presence | EN | II | I & II | - |
| Mobulidae | *Mobula diabolus* | *Mobula mobular* | Devil Ray | Confirmed presence | EN | II | I & II | - |
| Mobulidae | *Mobula mobular* | *Mobula mobular* | Devil Ray | Confirmed presence | EN | II | I & II | - |
| Mobulidae | *Mobula tarapacana* | *Mobula tarapacana* | Smoothtail Mobula | Confirmed presence | EN | II | I & II | - |
| Mobulidae | *Mobula thurstoni* | *Mobula thurstoni* | Ocellated Eagle Ray | Confirmed presence | EN | II | I & II | - |
| Myliobatidae | *Aetomylaeus maculatus* | *Aetomylaeus maculatus* | Mottled Eagle Ray | Confirmed presence | EN | - | - | - |
| [Myliobatidae](https://www.fishbase.se/summary/FamilySummary.php?ID=22) | *Aetomylaeus milvus* | *Aetomylaeus milvus* | Brown Eagle-Ray | Need further taxonomic work | EN | - | - | - |
| Myliobatidae | *Aetomylaeus nichofii* | *Aetomylaeus nichofii* | Banded Eagle Ray | Confirmed presence | VU | - | - | II |
| Narcinidae | *Narcine brevilabiata* | *Narcine brevilabiata* | Shortlip Electric Ray | Need further confirmation | VU | - | - | - |
| [Narcinidae](https://www.fishbase.se/summary/FamilySummary.php?ID=503) | *Narcine atzi* | *Narcine atzi* | Oman Numbfish | Northern Indian Ocean; patchy, Gulf of Oman, Bay of Bengal and Andaman Sea. | DD | - | - | - |
| Narcinidae | *Narcine brunnea* | *Narcine brunnea/timlei* | Brown Numbfish | Not a separate sp. Synonym of Narcine timlei | NE | - | - | - |
| [Narcinidae](https://www.fishbase.se/summary/FamilySummary.php?ID=503) | *Narcine lingula* | *Narcine lingula* | Chinese Numbfish | Indo–West Pacific | VU | - | - | - |
| Narcinidae | *Narcine maculata* | *Narcine maculata* | Darkfinned Numbfish | Confirmed presence | VU | - | - | - |
| Narcinidae | *Narcine prodorsalis* | *Narcine prodorsalis* | Tonkin Numbfish | Confirmed presence | DD | - | - | - |
| Narcinidae | *Narcine sp.* | *Narcine sp.* | Andaman Numbfish | Confirmed presence, potential new species to science. Further taxonomic work is needed | - | - | - | - |
| Narcinidae | *Narcine timlei* | *Narcine timlei* | Spotted Numbfish | Confirmed presence | DD | - | - | - |
| Narkidae | *Narke dipterygia* | *Narke dipterygia* | Numbray | Confirmed presence | DD | - | - | - |
| Pristidae | *Pristis cuspidatus* | *Anoxypristis cuspidata* | Pointed Sawfish | Confirmed presence | EN | I | I & II | I |
| Pristidae | *Pristis microdon/ Pristis pristis* | *Pristis pristis* | Largetooth Sawfish | Confirmed presence | CR | I | I & II | I |
| Pristidae | *Pristis pactinatus* | *Pristis pectinata* | Smalltooth Sawfish | Questionable record as distribution through Atlantic and South-West Indian Oceans; once widespread | CR | I | I & II | - |
| Pristidae | *Pristis zijsron* | *Pristis zijsron* | Longcomb Sawfish | Confirmed presence | CR | I | I & II | I |
| Rhinidae | *Rhina ancylostoma* | *Rhina ancylostoma* | Bowmouth Guitarfish | Confirmed presence | CR | II | - | - |
| Rhinidae | *Rhynchobatus djeddensis* | *Rhynchobatus djiddensis* | Giant Guitarfish | Distributed through Western Indian Ocean; South Africa to Oman. Possibly misidentified for *Rhynchobatus laevis* | CR | II | - | I |
| Rhinidae | *Rhynchobatus laevis* | *Rhynchobatus laevis* | Smoothnose Wedgefish | Confirmed presence | CR | II | - | - |
| [Rhinidae](https://www.fishbase.in/summary/FamilySummary.php?ID=713) | *Rhynchobatus australiae* | *Rhynchobatus australiae* | Bottlenose Wedgefish | Possible presence according to Kyne et al., 2020. Indo–West Pacific; Mozambique to eastern Australia. | CR | II | II |  |
| Glaucostegidae | *Rhinobatos typus* | *Glaucostegus typus* | Giant Shovelnose Ray | Confirmed presence | CR | II | - | - |
| Glaucostegidae | *Glaucostegus* cf. *granulatus* | *Glaucostegus* cf. *granulatus* | - | Confirmed presence, potential new species. | - | - | - | - |
| Glaucostegidae | *Rhinobatos granulatus* | *Glaucostegus granulatus* | Granulated Guitarfish | Confirmed presence | CR | II | - | I |
| Glaucostegidae | *Rhinobatus obtusus* | *Glaucostegus obtusus* | Blunt Shovel Nose Ray, Grey Guitarfish, Widenose Guitarfish | Confirmed presence | CR | II | - | - |
| Glaucostegidae | *Rhinobatos thouini* | *Glaucostegus thouin* | Thouin Ray | Confirmed presence | CR | II | - | - |
| Rhinobatidae | *Rhinobatos annandalei* | *Rhinobatos annandalei* | Annandale's Guitarfish | Confirmed presence | DD | - | - | - |
| Rhinobatidae | *Rhinobatos lionotus* | *Rhinobatos lionotus* | Smoothback Guitarfish | Confirmed presence | DD | - | - | - |
| Rhinobatidae | *Rhinobatos ranongensis* | *Rhinobatos ranongensis* | Ranong guitarfish | Confirmed presence | NE | - | - | - |
| Rhinopteridae | *Rhinoptera bonasus* | *Rhinoptera bonasus* | American cownose Ray | Questionable record as distribution through Western Atlantic; New England (USA) to northern Argentina | VU | - | - | - |
| Rhinopteridae | *Rhinoptera adspersa* | *Rhinoptera javanica* | Flapnose Ray | Confirmed presence | VU | - | - | - |
| Rhinopteridae | *Rhinoptera javanica* | *Rhinoptera javanica* | Flapnose Ray | Confirmed presence | VU | - | - | - |
| Rhinopteridae | *Rhinoptera jayakari* | *Rhinoptera jayakari* | Oman Cownose Ray | Confirmed presence | NE | - | - | - |
| [Rajidae](https://www.fishbase.se/summary/FamilySummary.php?ID=19) | *Dipturus johannisdavisi* | *Dipturus johannisdavisi* | Travancore Skate | Bay of Bengal is a range according to the maps of Last et al., 2016. Indian Ocean; Bay of Bengal and India, possibly west to Tanzania. Possible presence | DD | - | - | - |
| [Rajidae](https://www.fishbase.se/summary/FamilySummary.php?ID=19) | *Orbiraja powelli* | *Orbiraja powelli* | Indian Ring Skate | Northern Indian Ocean; Arabian Sea (India) to Bay of Bengal (Myanmar). Possible presence | NT | - | - | - |
