# Supplementary material for: Evaluating artisanal fishing of globally threatened sharks and rays in the Bay of Bengal, Bangladesh
Source: PLoS One. 2021 Sep 9;16(9):e0256146. doi: 10.1371/journal.pone.0256146 (PMC8428726; doi:10.1371/journal.pone.0256146)
Supplement: S2 Table — (DOCX) [file pone.0256146.s005.docx]

**S2 Table.** Relative landings of aggregate elasmobranchs in the Bay of Bengal countries from 1950-2016 with and mean, standard deviation and percentage of total catch contribution from the Bay of Bengal areas.

| Country | Total landing (1950-2016) t | Mean ± SD | % | Landing (1990-2016) t | Mean ± SD | % |
| --- | --- | --- | --- | --- | --- | --- |
| Bangladesh | 330720 | 4936.12± 3186.92 | 3.32 | 213100 | 7892.59± 1910.83 | 4.00 |
| India | 3643840 | 54385.67± 21282.76 | 36.61 | 1868800 | 69214.81± 16998.18 | 35.11 |
| Indonesia (Indian Ocean) | 1699080 | 25359.40± 19063.46 | 17.07 | 1210240 | 44823.70± 9038.45 | 22.74 |
| Malaysia (Peninsula West) | 652680 | 9741.49± 5658.57 | 6.56 | 320560 | 11872.59± 2669.55 | 6.02 |
| Thailand (Andaman Sea) | 289060 | 4314.33± 2119.33 | 2.90 | 139210 | 5155.93± 2513.09 | 2.62 |
| Thailand (Gulf) | 535000 | 7985.07± 3365.15 | 5.38 | 221090 | 8188.52± 4094.56 | 4.15 |
| Myanmar | 1917140 | 28614.03± 11396.72 | 19.26 | 1021830 | 37845.56± 11917.88 | 19.20 |
| Sri Lanka | 821370 | 12259.25± 4806.44 | 8.25 | 304990 | 37845.56± 11917.88 | 5.73 |
| Maldives | 64530 | 963.13± 668.22 | 0.65 | 22510 | 11295.93± 4812.09 | 0.4 |
| Total | 9953420 | 148558.51± 53762.66 | 100 | 5322330 | 833.70± 540.41 | 100 |
