# Supplementary material for: Evaluating artisanal fishing of globally threatened sharks and rays in the Bay of Bengal, Bangladesh
Source: PLoS One. 2021 Sep 9;16(9):e0256146. doi: 10.1371/journal.pone.0256146 (PMC8428726; doi:10.1371/journal.pone.0256146)
Supplement: S3 Table — (DOCX) [file pone.0256146.s006.docx]

**S3 Table.** Observation on the abundance of different elasmobranch species in landing, plausible reasons and conservation implications.

| Species | Abundance in landing | Reasons and conservation implications |
| --- | --- | --- |
| Sharks | | |
| *S. laticaudus* | Very common | The most commonly documented shark species was the spadenose shark, *S. laticaudus*, consistent with previous findings [1]. The mortality of this species is high in this region at 1.31 yr-1 and fishing mortality of 0.745 yr-1 [1, 2]. This is presumably due to relatively high fecundity, its occurrence in shallow water (13 m) demersal habitats [3], which frequently are exploited by a substantial number of artisanal boats deploying a substantial number of gears [4]. However, the value of these sharks in the landing site was less than larger sharks due to no international demand for fin trade. |
| *Chiloscyllium* spp. | Moderately common | Chiloscyllium spp. were also of less value, still landed in high numbers. This is presumably due to the practice of less discard in this region, and any value is vital for the fishers, no matter how small. At least six species of this genus are present in this region (e.g. Chiloscyllium hasselti, C. burmensis, C. indicum, C. punctatum, C. griseum, C. cf. arabicum) [5-7, Haque in review], however four species were reported during this study with one species needing further taxonomic identification (C. cf. arabicum) for confirmation. Since many gears in this region target demersal species [8], the catch of these species is quite prevalent. |
| *C. sorrah, G. cuiver, C. ambionensis* | Common | Carcharhinids were the most abundant sharks reported in this study; after spadenose shark especially spottail shark C. sorrah, blacktip shark C. limbatus, tiger shark G. cuiver and pigeye sharks C. ambionensis followed by bull shark C. leucas. All these species were landed in abundance at all sites and are reported to be extremely valuable because of the fin trade [8]. These are a mix of freshwater, estuarine, reef-associated and pelagic species indicating the diverse nature of artisanal fisheries. These species are highly desired amongst the fishers and traders and increased retention and target with hooks. Although modified gill nets to target sharks have been reported [2] targeting larger sharks. However, this practice decreased over time, and during the study period, no substantial number of such practices were observed (Haque unpubl. data). Carcharhinids are highly important for both artisanal and commercial fisheries worldwide, contributing largely to marine fisheries landings [9-13]. |
| *C. limbatus, C. leucas* | Moderately common |  |
| *Carcharhinus falciformis, C. amblyrhynchoides, C. brevipinna and C. macloti* | Uncommon | Although Carcharhinus falciformis, C. amblyrhynchoides, C. brevipinna and C. macloti were not abundantly reported, these were historically reported in greater numbers [7]. This is either due to depleted population in this region or sampling bias as smaller sharks in bigger piles of more than 500 individuals could not be identified; however, that is less likely, and most of them were spadenose sharks. |
| *Lamiopsis temmincki* | Very uncommon | Although *Lamiopsis temmincki*was reported in Cox’s Bazar previously [3], in this study, it was opportunistically sampled in two landing sites and was absent in St. Martin’s Island. |
| *Stegostoma fasciatum* | Rare | *Stegostoma fasciatum* was recorded just once in this study whereas it was reported more frequently previously [2]. |
| Hammerhead sharks | *S. lewini* is common, others are rare | The scalloped hammerhead shark was very commonly caught at all landing sites, including juveniles, and is extremely desirable to the fishers and traders due to the high value of fins [8]. Although other hammerhead sharks were previously reported from this region (e.g. winghead shark E. blochi, great hammerhead shark, S. mokarran) [7, 14], they were not recorded in the current study. This discrepancy may be due to an extreme E. blochi, S. mokarran and Sphyrna zygaena population decline in the region. This is a serious conservation concern as now the pressure is entirely on S. lewini, an Endangered species, and a global decline in populations is reported [15]. The demand is due to the larger size of the fins and the overall larger size of the shark providing comparatively higher value. |
| Thresher sharks |  | Consistent with our findings, Thresher sharks (family: Alopidae) are rarely reported within the Bay of Bengal, Bangladesh. Two species (e.g. A. pelagicus and A. supercilious) were previously reported from longlines and drift gill nets surveying pelagic waters [16]. The one Alopidae specimen recorded was identified to genus Alopias sp. based on a characteristic feature in this family of sharks, the elongated caudal fin [12]. The lack of reports is either since these species are highly pelagic and the range does not overlap with artisanal fisheries except for rare occasions |
| Rays | | |
| *G. poecilura* | Very common | **The most commonly found ray was G. poecilura, consistent with previous studies. Two species of butterfly rays have been reported from Bangladesh (G. japonica and G. poecilura) previously [7, 14]. The presence of different spot patterns and morphs that have been documented may be due to geographic population differences; however, none was confirmed to be G. japonica. The traders moderately value these. The number could be higher due to higher breeding potential than many other elasmobranch species [17]. The number of juveniles encountered was highest in the winter and pre-monsoon season, probably due to the presence of inshore nursery grounds [17].** |
| *Neotrygon spp.* | Very common | The second highest species sampled belonged to the genus Neotrygon.  Several species have been recently described under this genus (Neotrygon australiae, Neotrygon caeruleopunctata, Neotrygon orientale, Neotrygon trigonoides, Neotrygon bobwardi, Neotrygon malaccensis, Neotrygon moluccensis, Neotrygon westpapuensis, Neotrygon vali) [18-21] three variants morphologically most consistent with N. kuhlii. N. indica and N. caeruleopunctata were documented. The population variants of Neotrygon spp. in the Bay of Bengal need further taxonomic work as differentiation has been observed between populations [18]. All of these species are either reef associated or demersal inshore species hence overlap with artisanal bottom net fisheries and longline hooks. Hence the number landed were substantially high, although the demand was comparatively low from other bigger rays. |
| *Himantura spp.* | Very common | Amongst bigger rays the present study has documented three species under the genus Himantura (H. leoparda, H. undulata and H. uarnak). Recently, a new ocellated whipray Himantura tutul has been described using DNA barcoding [18], which has been described as a synonym for H. uarnak by Last et al., 2016. As a result, this has not been added to the species list, although morphologically similar specimens were documented. Species belonging to this family are highly valued, not only for meat consumption [21] but also for the quality of the skin used as a raw material for accessories worldwide [21]. |
| *Maculabatis spp., Brevitrygon spp., and Pateobatis spp., Urogymnus polylepis.* | Moderately common | Maculabatis, Brevitrygon, and Pateobatis were moderately commonly observed, followed by Urogymnus. Species from the genus Pastinachus, Telatrygon were rarely recorded. These rays are bottom dwellers [21] and are heavily exploited by the non-baited long lines targeting rays in these waters; however, the uncommon nature of landing could not be corroborated with any indication of population decline due to the absence of historical data. |
| *Pastinachus spp., Telatrygon spp.* | Uncommon |  |
| Devil rays, Cownose rays and Eagle rays | Moderately common | **Devil rays, Cownose rays and Eagle rays** were also moderately abundant throughout the study period, especially M. mobular, R. javanica, R jayakari and A. oscellatus. Although these species are mostly pelagic or benthopelagic (rhinopteridae), inshore occurrences are not rare [22]. Devil rays are valuable to the traders due to the international demand of gill rakers [22-24]. Whereas Mobula mobular was commonly encountered, M. eregoodoo and M. birostris were rarely observed, probably due to being highly pelagic or naturally rare in this region. |
| *Narcine spp. and Narke sp.* | Uncommon | The three numbfishes and one sleeper ray reported in this study were extremely rare in this region. Although reported to be found relatively commonly previously [6, 25], the capture of these species was low in number in this study. In some cases, electric ray species with less market demand and values, such as Narcine spp., might have been discarded at sea due to the fear of being electrocuted (Haque, pers. comm. fishers, January 2020) and thus are presumably not accurately represented in landings. |
| Rhinopristoformes rays | | |
| *G. granulatus and G. obtusus followed by R. ancylostoma* | Common | **Rhinopristoformes rays were** abundantly landed, especially G. granulatus and G. obtusus, followed by R. ancylostoma. These species are frequently targeted using non-baited long lines due to high fin price and meat consumption [26]. Despite frequent landings of these species, owing to both target and non-discarded/opportunistic fishing practices, little is known about their geographic distribution and species composition in the North Indian Ocean [26]. Although G. granulatus, R. ancylostoma, G. typus, and R. djiddensis (probably R. lavies or R. australie, as R. djiddensis does not occur in this region) were commonly previously reported [2, 7, 25], the current study reported no Rhynchobatas spp., presumably due to extreme population decline. A potential population depletion is corroborated by fishers, commonly referred to as a white-spotted guitarfish, which is not found anymore (Haque unpubl. data); however, a more comprehensive investigation is required to confirm this. The depletion of the Rhinopristoformes ray population in the wider Indo-Pacific is well documented, whereby 80-99% of the population declined throughout their range due to overfishing [26]. The scenario is similar in Bangladesh. Previous records documented substantially high landings of these species with a total of 10.448 MT of R. djiddensis (probably R. australiae and R. laevis) along with ~30 MT of G. granulatus landed in only two sites across coastal Bangladesh [27]. A continuous landing was reported for R. laevis from 2006 to 2012 [28].  Additionally, between 2011-12 a total of 2.30 MT of R. laevis along with 0.09 MT of R. ancylostoma and 27.16 MT of R. typus (presently G. typus) [27] were landed. However, the composition in landing has changed drastically now, whereby only G. granulatus and G. obtusus are currently observed in abundance, and all other species have substantially decreased. This is an early warning of extreme population decline and the pressure being replaced on G. granulatus and G. obtusus as they are extremely valuable in the international fin trade [29, Haque in review]. If fisheries like this continue to occur at this rate, Bangladesh may see a complete collapse of Rhinopristoformes rays very soon. Proactive measure to manage these fisheries is crucial and highly recommended. |
| *G. typus, R.* *lionatus*, and *R. annadalei* | Very uncommon |  |
| *Rhynchobatas spp.,* | Rare |  |
| *Pristis spp.* | Rare | It is likely that the occurrence of extremely valuable rays, such as the largetooth sawfish, is unreported in most recent studies as incidental by-catch does not land in the formal landing sites. However, throughout the study period, a total of 33 largetooth sawfish was also recorded, indicating the landing is higher than documented previously by Haque in press 2020. Although three sawfish species, the largetooth, green and narrow sawfish, were documented in a former study in Bangladesh [30-33], the current study did not document any recent landings of narrow sawfish. However, in 2006, three specimens of narrow sawfish were documented in Chittagong [33], and 1.145 MT of narrow sawfish landings were reported between 2006 and 2010 [27]. The most recent study by Hossain et al. 2015, mentioned 53 sawfish ladings in Cox's Bazar from BFRI between 2010-2011, all of which were mistakenly identified as A. cuspidata; however, no landings of narrow sawfish were actually documented [30] consistent with our study. This could be either because of data bias, as the study could not focus on the south central and south western coastal areas, or a very high possibility of a very steep decline of the population of sawfishes. The latter's possibility is stronger as the narrow sawfish has been reported to be 'rarely found' in Bangladesh waters (7), and the green sawfish has been confirmed for the first time [31]. As a result, there has not been a confirmed record of narrow sawfish from Bangladesh waters since 2010. Even in India, narrow sawfish' landing has significantly reduced from 1989 to 2011 [34]. However, a study conducted between 2010 and 2013 likely reported 19 sawfish individuals, wrongly naming it 'saw shark Pristis cuspidatus' [2] (though confirmation could not be made). |

**References for S3 Table**

1. Karim E, Qun LI, Mahmood MA, Baset A, Hoq ME, Shamsuzzaman MM, Das A. Assessment of some demographic trends of Spadenose shark (Scoliodon laticaudus) of the Bay of Bengal, Bangladesh. 2017.
2. Karim E, Zaher M, Barua S, Rahman MJ, Hoq E. Catch composition, seasonal abundance and length-weight relationship of elasmobranch species of the Bay of Bengal, Bangladesh. Banglad. J. Fish Res 2012;15:115-24.
3. Froese R, Pauly D. FishBase. World Wide Web electronic publication. 2009 [cited 2021 Feb 7]. Available from: http://www. fishbase. org.
4. Shamsuzzaman MM, Islam MM, Tania NJ, Al-Mamun MA, Barman PP, Xu X. Fisheries resources of Bangladesh: Present status and future direction. Aquac Fish. 2017 Jul 1;2(4):145-56.
5. Ahmed MS, Chowdhury NZ, Datta SK, Zhilik AA. New geographical record of the Burmese bamboo shark, Chiloscyllium burmensis (Orectolobiformes: Hemiscylliidae), from Bangladesh waters. Thalassas. 2019 Oct;35(2):347-50.
6. Ahmed MS, Datta S, Saha T, Hossain Z. Molecular characterization of marine and coastal fishes of Bangladesh through DNA barcodes. Authorea [Preprints]. 2020 Jun 11 [cited 2021 February 7]. Available from: https://www.authorea.com/users/332255/articles/458700-molecular-characterization-of-marine-and-coastal-fishes-of-bangladesh-through-dna-barcodes.
7. Hoq ME, Haroon AY, Hussain MG, editors. Shark fisheries in the Bay of Bengal, Bangladesh: Status and potentialities. Support to Sustainable Management of the BOBLME Project, Bangladesh Fisheries Research Institute; 2011 Jan.
8. Haque AB, Biswas AR, Latifa GA. Observations of shark and ray products in the processing centres of Bangladesh, trade in CITES species and conservation needs. TRAFFIC Bulletin. 2018;30(1):6-14.
9. Castillo-Géniz JL, Márquez-Farias JF, De La Cruz MR, Cortés E, Del Prado AC. The Mexican artisanal shark fishery in the Gulf of Mexico: towards a regulated fishery. Mar Freshw Res. 1998;49(7):611-20.
10. Compagno LJ, Dando M, Fowler S. Sharks of the World. 2005. p. 368.
11. Henderson AC, Reeve AJ, Jabado RW, Naylor GJ. Taxonomic assessment of sharks, rays and guitarfishes (Chondrichthyes: Elasmobranchii) from south-eastern Arabia, using the NADH dehydrogenase subunit 2 (NADH2) gene. Zool J Linnean Soc. 2016 Feb 1;176(2):399-442.
12. White WT, Dharmadi. Species and size compositions and reproductive biology of rays (Chondrichthyes, Batoidea) caught in target and non‐target fisheries in eastern Indonesia. J Fish Biol. 2007 Jun;70(6):1809-37.
13. Last PR, Stevens JD. Sharks and rays of Australia. 2009.
14. Roy BJ, Singha NK, Rhaman MG, Ali AH. Status and recorded of sharks and rays in the Bay of Bengal of Bangladesh Region. Braz J Biol Sci. 2015 Dec 31;2(4):343-67.
15. [The IUCN Red List of Threatened Species. Version 2020-3 [cited 2021 Feb 7]. In; IUCN Redlist website [Internet]. Available from: https://www.iucnredlist.org.](https://www.iucnredlist.org/)
16. Krajangdara T. New Record of Cartilaginous Fishes Found in Thai Waters and the Adjacent Areas and an Updated Species List in 2019. Burapha Sci J (วารสาร วิทยาศาสตร์ บูรพา). 2019 May 16;24(2):599-621.
17. Raje SG, Zacharia PU. Investigations on fishery and biology of nine species of rays in Mumbai waters. Indian J Fish. 2009;56(2):95-101.
18. Borsa P, Arlyza IS, Chen WJ, Durand JD, Meekan MG, Shen KN. Resurrection of New Caledonian maskray Neotrygon trigonoides (Myliobatoidei: Dasyatidae) from synonymy with N. kuhlii, based on cytochrome-oxidase I gene sequences and spotting patterns. C R Biol. 2013 Apr 1;336(4):221-32.
19. Borsa P, Arlyza IS, Hoareau TB, Shen KN. Diagnostic description and geographic distribution of four new cryptic species of the blue-spotted maskray species complex (Myliobatoidei: Dasyatidae; Neotrygon spp.) based on DNA sequences. J Oceanol Limnol. 2018 May;36(3):827-41.
20. Borsa P. Neotrygon vali, a new species of the blue-spotted maskray complex (Myliobatoidei: Dasyatidae). bioRxiv. 2017 Jan 1:106682.
21. Last PR, White WT, Seret B. Taxonomic status of maskrays of the Neotrygon kuhlii species complex (Myliobatoidei: Dasyatidae) with the description of three new species from the Indo-West Pacific. Zootaxa. 2016 Feb 23;4083(4):533-61.
22. Camhi MD, Valenti SV, Fordham SV, Fowler SL, Gibson C. The conservation status of pelagic sharks and rays: report of the IUCN shark specialist group pelagic shark red list workshop. IUCN Species Survival Commission Shark Specialist Group. Newbury, UK; 2009. p. 78.
23. Croll DA, Dewar H, Dulvy NK, Fernando D, Francis MP, Galván‐Magaña F, Hall M, Heinrichs S, Marshall A, Mccauley D, Newton KM. Vulnerabilities and fisheries impacts: the uncertain future of manta and devil rays. Aquat Conserv. 2016 Jun;26(3):562-75.
24. Zeng Y, Wu Z, Zhang C, Meng Z, Jiang Z, Zhang J. DNA barcoding of mobulid ray gill rakers for implementing CITES on elasmobranch in China. Sci Rep. 2016 Nov 23;6(1):1-9.
25. Hoq ME, Haroon AY. Sharks, Skates & Rays of Bangladesh. Support to Sustainable Management of the BOBLME Project, Bangladesh Fisheries Research Institute. 2014.
26. Kyne PM, Jabado RW, Rigby CL, Gore MA, Pollock CM, Herman KB, Cheok J, Ebert DA, Simpfendorfer CA, Dulvy NK. The thin edge of the wedge: extremely high extinction risk in wedgefishes and giant guitarfishes. Aquat Conserv. 2020 Jul;30(7):1337-61.
27. Roy BJ, Alam MF, Rhaman MG, Singha NK, Akhtar A. Landing trends, species composition and percentage composition of Sharks and Rays in Chittagong and Cox‟ s Bazar, Bangladesh. Glob. J Sci Front Res. 2014;14:070403-9999.
28. Roy BJ, Singha NK, Ali SH, Rhaman MG. Availability of vulnerable elasmobranches in the marine water of Bangladesh. Bangladesh J Zool. 2012;40(2):221-9.
29. Moore AB. Are guitarfishes the next sawfishes? Extinction risk and an urgent call for conservation action. Endanger Species Res. 2017 Jul 21;34:75-88.
30. Hossain MA, Thompson BS, ChowdHury GW, Mohsanin S, Fahad ZH, Koldewey HJ, Islam MA. Sawfish exploitation and status in Bangladesh. Aquat Conserv. 2015 Dec;25(6):781-99.
31. Haque AB, Das SA. New records of the Critically Endangered Ganges shark Glyphis gangeticus in Bangladeshi waters: urgent monitoring needed. Endanger Species Res. 2019 Oct 17;40:65-73.
32. Haque AB, Leeney RH, Biswas AR. Publish, then perish? Five years on, sawfishes are still at risk in Bangladesh. Aquat Conserv. 2020 Dec;30(12):2370-83.
33. Roy BJ, Dey MP, Alam MF, Singha NK. Status of shark fishing in the marine water of Bangladesh. UNEP/CMS/MS/Inf/10; 2007.
34. Bineesh KK, Moore AB, Kyne PM. Indo-west pacific, Chapter 7. Sawfish: a global conservation category. IUCN Species Survival Commission’s Shark Specialist Group, Vanc. 2014;62.
